# Unraveling the *Plasmodium vivax* sporozoite transcriptional journey from mosquito vector to human host

**DOI:** 10.1038/s41598-018-30713-1

**Published:** 2018-08-15

**Authors:** Alison Roth, Swamy R. Adapa, Min Zhang, Xiangyun Liao, Vishal Saxena, Raaven Goffe, Suzanne Li, Ratawan Ubalee, Gagandeep S. Saggu, Zarna R. Pala, Shilpi Garg, Silas Davidson, Rays H. Y. Jiang, John H. Adams

**Affiliations:** 1 Center for Global Health and Infectious Diseases Research, College of Public Health, University of South Florida, Tampa, Florida USA; 2 Molecular Parasitology and System Biology Lab, Department of Biological Sciences, Birla Institute of Technology and Science, Pilani, Rajasthan India; 3 Department of Entomology, Armed Forces Research Institute of Medical Sciences, Bangkok, Thailand; 4 Laboratory of Malaria and Vector Research, National Institute of Allergic and Infectious Diseases, National Institute of Health, Rockville, Maryland USA

## Abstract

Malaria parasites transmitted by mosquito bite are remarkably efficient in establishing human infections. The infection process requires roughly 30 minutes and is highly complex as quiescent sporozoites injected with mosquito saliva must be rapidly activated in the skin, migrate through the body, and infect the liver. This process is poorly understood for *Plasmodium vivax* due to low infectivity in the *in vitro* models. To study this skin-to-liver-stage of malaria, we used quantitative bioassays coupled with transcriptomics to evaluate parasite changes linked with mammalian microenvironmental factors. Our *in vitro* phenotyping and RNA-seq analyses revealed key microenvironmental relationships with distinct biological functions. Most notable, preservation of sporozoite quiescence by exposure to insect-like factors coupled with strategic activation limits untimely activation of invasion-associated genes to dramatically increase hepatocyte invasion rates. We also report the first transcriptomic analysis of the *P. vivax* sporozoite interaction in salivary glands identifying 118 infection-related differentially-regulated *Anopheles dirus* genes. These results provide important new insights in malaria parasite biology and identify priority targets for antimalarial therapeutic interventions to block *P. vivax* infection.

## Introduction

Malaria infections are initiated when a *Plasmodium-*infected *Anopheles* mosquito bites a mammalian host, injecting saliva-laden sporozoites into the skin^1–5^. Before transmission, salivary gland sporozoites can remain in a quiescent state for days to weeks under ideal environmental conditions. However, once sporozoites are injected into the skin, a just-in-time activation of molecular processes and induction of gene expression stimulates a remarkably efficient process whereby sporozoites make a ‘mad dash’ to reach the liver and infect hepatocytes^6^. The initial sporozoite activation appears to be linked to the drastic changes in the newly encountered microenvironment, such as change in pH, temperature, amino acids, and serum proteins, especially albumin^7–9^. However, replicating the *in vivo* infection processes in the laboratory is inherently difficult as studies typically begin with isolation of sporozoites through dissection of salivary glands thus resulting in dramatically reduced sporozoite infection rates in cultured hepatocytes compared to infections initiated by direct mosquito bite^10^.

Activation in the skin represents the initial phase of the malaria infection process and it is thought that sporozoites are progressively activated by refined cellular mechanisms to recognize and then respond to extrinsic microenvironmental factors for motile migration, cell traversal, and ultimately hepatocyte invasion^11–13^. A better understanding of the molecular activations that occur during infection should help improve *in vitro* systems to more closely reflect *in vivo* outcomes. Sporozoite motility in the skin is important for parasites to rapidly enter human blood vessels enabling transport to the liver sinusoid by blood circulation. Once in the liver, sporozoites rely on cell traversal to passage through and eventually invade a host hepatocyte^14,15^. While entry into the circulatory system can be immediate, studies have shown accumulation of sporozoites at the injection site for up to 42 hours with almost half of the inoculation present after 3 hours^1,2,16^. Consistent with the just-in-time requirement for sporozoite activation, prolonged *in vitro* exposure of *Plasmodium falciparum* sporozoites to human body temperature, serum proteins, primary human hepatocytes (PHHs), or human skin keratinocytes have been shown to prematurely transform sporozoites to early liver-stage parasites leading to reduction in sporozoites infectivity^9,17^.

*Plasmodium vivax*, the focus of this study, is less well-characterized but is the widest geographically-distributed malaria parasite placing over 2.85 billion people at risk of infection^18–21^. Unlike other human malarias, *P. vivax* has a unique fundamental biological feature where the parasite can remain clinically dormant as a hypnozoite inside the liver until unknown reactivation mechanisms cause relapse to blood-stage^22,23^. Recently, there has been a renewed interest in *P. vivax* due to reports of *P. vivax* dominance in areas previously dominant for *P. falciparum* and the prevalence of both asymptomatic and sub-microscopic parasite carriers^24–27^. However, the inability to maintain continuous *in vitro* blood-stage culture in the laboratory causes sporozoites usage for studies on *P. vivax* vector interactions and liver-stage biology to primarily rely on resource-intensive mosquito infections from clinical isolates. Therefore, a critical need exists to improve efficiency of sporozoite usage to support studies on the unique biology of this neglected malaria parasite, especially to develop new interventions to prevent relapse from dormant liver-stage parasites.

There are more than 400 anopheline mosquito species in the world with over 40 known as dominant vectors for malaria-causing *Plasmodium* species^28^. *Anopheles* mosquito vectors are more permissive to *P. vivax* gametocyte infection than *P. falciparum* showing highly effective transmission rates in far more diverse climates^20,29^. In continental Southeast (SE) Asia, 19 dominant vector species are present, where *Anopheles dirus sensu lato* (s.l.) (Dirus Complex), *An. minimus s.l*. (Minimus Complex), and *An. sundaicus s.l*. (Sundaicus Complex) present as the three most competent malaria vectors. Within the Dirus complex, *An. dirus* (*An. dirus sensu stricto*) is historically the dominant malaria vector in Thailand, Laos, Cambodia, and Myanmar feeding primarily on humans with suitable adaption to human-induced environmental changes^30^. New approaches for entomological malaria transmission metrics are needed for elimination, particularly in regions with diverse vectors and low parasite densities^31^. Identifying *P. vivax* or vector-specific biomarkers in mosquito saliva would improve estimates of transmission variables, and such biomarkers may also serve as targets for transmission interruption^32,33^.

In this study, we report progressive gene activation in *P. vivax* sporozoites in response to microenvironmental stimuli that mimic host factors encountered when transitioning from mosquito to mammal. Effects on sporozoites by modification of the microenvironment were assessed using biometric measurements from *in vitro* functional assays targeting sporozoite motility and invasion of PHHs. These functional assays paired with RNA-seq reveal changes in gene expression associated with sporozoite infectivity and initiation of early liver-stage parasite development. Most importantly, we demonstrate that strategic manipulation of the sporozoite microenvironment can significantly enhance sporozoite *in vitro* infectivity for cultured hepatocytes. Furthermore, using our salivary gland isolation techniques we identified transcriptional signatures in the *An. dirus* mosquito harboring *P. vivax* sporozoites implicating dynamic biological variables that may affect transmission of *P. vivax*. Overall, this study reveals rapid transcriptional changes in *P. vivax* sporozoites in response to host-like stimuli as well as in the *An. dirus* mosquito vector that are important regulators affecting infectivity and transmission.

## Results

### Selection of media and study design to emulate insect and human host-like microenvironment

**Figure 1:**
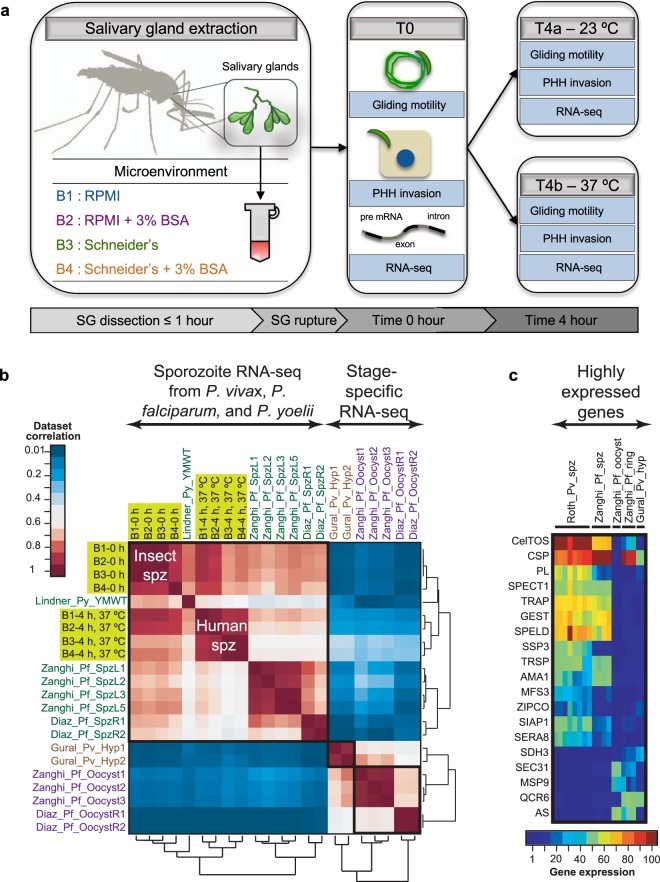
Experimental design for RNA-sequencing of *P. vivax* salivary gland sporozoites (PvSGSs) and transcriptome comparison to different stage-specific *Plasmodium* species. (**a**) PvSGSs are aseptically dissected under an hour into respective experimental microenvironments (B1 = RPMI, B2 = RPMI + 3% BSA, B3 = Schneider’s, B4 = Schneider’s + 3% BSA) with no requirement of purification. Time point 0 h begins immediately after mosquito dissections where PvSGSs are measured in a gliding motility assay and a primary human hepatocyte invasion (PHH) assay with samples simultaneously collected for RNA-seq. This process is repeated for 2 additional time points; (4 h post-dissection at RT and 4 h post-dissection at 37 °C). (**b**) Sporozoites transcriptomes show overall similarities between *P. vivax, P. falciparum*, and *P. yoelii*. Different sporozoites transcriptomes generated in different labs in different species show overall similarities with Kendall’s tau >0.5; and they are less similar to other liver-stage related datasets, like hypnozoite transcriptomes, and oocytes transcriptomes. The datasets generated in this study are shaded yellow. The dendrographs represent the dataset relationships based on correlation of orthologue expression levels. (**c**) Orthologous gene expression levels in *Plasmodium* liver-stage parasites. Sporozoites enriched genes like CelTos, SPECT1, and TRAP are highly expressed in sporozoites in different *Plasmodium* species. Gene expression levels were normalized within sample as expression level ranks for comparisons.

The microenvironment composition was selected based on media components previously determined to resemble mosquito and human host-like environments that salivary gland sporozoites encounter upon transmission^34,35^. Schneider’s insect media (pH 7) was used to mimic the mosquito microenvironment whereas incomplete RPMI 1640 media with the addition of 3% (w/v) of bovine serum albumin (BSA) represented the human microenvironment (Supplementary Table S1). Our study design focused on the serum protein albumin as it is the most abundant (>50%) of total serum proteins and has important roles in transport as well as modulating osmotic pressure^36^. Additionally, previous studies have shown that addition of albumin significantly contributes to sporozoite activation^8,35^. Further, we used BSA rather than human serum albumin as they are chemically similar, and to avoid serum-donor bias and non-specific protein binding. In total, four different buffered media compositions were tested including RPMI (B1), RPMI + 3% BSA (B2), Schneider’s (B3), and Schneider’s + 3% BSA (B4) (Fig. 1a).

In order to recapitulate the sporozoite’s transitional journey from vector to host, our study design considered initial time zero (0 h) immediately after salivary gland isolation (manual dissection) with subsequent 4 h post-dissection incubations at two different temperatures, room temperature (RT) or 37 °C (Fig. 1a). We hypothesized that the prolonged 4 h post-dissection sampling would capture sporozoite gene expression specific to the microenvironment in which the sporozoite was maintained. For instance, transformation into early liver-stage parasites when held in a human-like microenvironment, as previously shown in Kaiser *et al*.^17^, or preservation of a quiescent transcriptional state when maintained in an insect-like microenvironment. Additionally, the 4 h post-dissection samples at RT or 37 °C were used to investigate the effect on the sporozoite when exposed to temporal temperature shift. Further, our salivary gland isolations typically consisted of 10–20 mosquitoes per microenvironment condition and required no additional sporozoite purification, reducing sample manipulation. Lastly, *P. vivax* salivary gland sporozoites (PvSGSs) in the various microenvironment conditions were phenotypically assessed at each time point using *in vitro* assays measuring percent gliding motility and invasion rate of PHHs (Fig. 1a).

All *P. vivax* patient isolate-derived samples (48 total) were analyzed for expression of genes showing low experimental variation indicated between replicates (Supplementary Fig. S1a). These samples collected for RNA-seq included 5 biological replicates (patient isolates) for 0 h post-dissection, 3 biological replicates at 4 h post-dissection kept at RT, and 4 biological replicates at 4 h post-dissection kept at 37 °C. The quantified transcript abundance of PvSGS-infected samples showed a mean of 14% ± 9% (± standard deviation.) mapped to *P. vivax* reference genome P01 (PlasmoDB, version 34), a mean of 50% ± 12% mapped to the WRAIR2 *An. dirus* reference genome (Vectorbase, VB-2018-02, AdirW1.7), and a mean of 36% ± 5% of unmapped reads (mean transcript count ≥20 FPKM) (Supplementary Fig. S1b). We achieved very high coverage of the *P. vivax* genome, where approximately 10–30% of the total reads (on average half a million malarial parasite reads per sample) are mapped back to the *P. vivax* reference sequences covering over 4000 genes with high confidence, confirming the high-quality preparation of our samples. Furthermore, an average of 70% of RNA transcripts detected from PvSGS samples and uninfected salivary gland controls mapped to *An. dirus* reference genome with, >93% of transcripts mapped to salivary gland-associated genes previously identified from an *An. gambiae* proteomic study^37^ (Supplementary Fig. S1c).

### Sporozoite transcriptome signatures linked to host microenvironments

Initially, sporozoite transcripts were identified through a comprehensive comparison of our sporozoites transcriptome to datasets previously published for *P. vivax* blood-stages^38^. We identified 3,510 up-regulated genes and 1,923 down-regulated genes in the sporozoite stage compared to blood-stages, of which 903 genes were identified as specific to sporozoites (Supplementary Fig. S2 and Supplementary Data S2). As a comparison of sporozoite to blood-stage gene expression across species, we additionally examined previously generated sporozoites transcriptome data from *P. falciparum*^39,40^, *P. vivax*^41^, and *P. yoelii*^42^ (Fig. 1b). The datasets were re-normalized to a scale from 1 to 100, and similarity was calculated using a non-parametric method (Kendall’s Tau). There are overall similarities between sporozoites data across different species with a conserved set of highly expressed genes, such as Cell Traversal protein for Ookinetes and Sporozoites (CelTOS) and Circumsporozoite Protein (CSP) (Fig. 1c). The overall correlation of transcriptome expression is stronger between the same stage of different species (i.e., sporozoite vs. sporozoite) than the correlation between different stages of the same species (i.e. oocyst or hypnozoite) (Fig. 1b).

**Figure 2:**
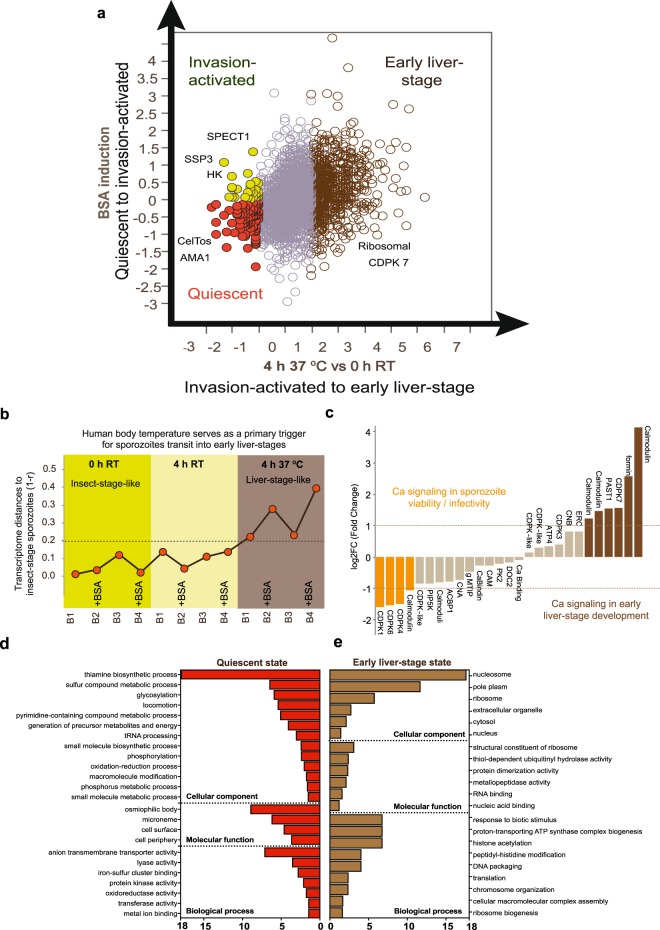
*P. vivax* salivary gland sporozoite (PvSGS) microenvironment linked to transcriptome signatures. **(a)** Three phenotypic groups were indicated in RNA-seq analysis, quiescent, invasion activated, and early liver-stage, which were determined through differential gene expression induced by serum component albumin and temperature shift from RT to 37 °C. **(b)** Pearson’s correlation of all RNA-seq samples shows temperature shift serves as the major trigger for PvSGSs transition to early liver-stage parasites. **(c)** Distinct calcium signaling cascades are identified as PvSGSs transition where calmodulin, CDPK 1, 4, 6 are influential in PvSGSs invasion with presence during quiescent state while calmodulins, formin, CDPK7, and PAST1 are implemented with roles in early liver-stage parasite development. **(d)** Gene Ontology (GO) enrichment analysis were performed on sporozoite quiescent state (red) and early liver-stage state (brown) with emphasis on cellular components, molecular function, and biological processes where bar graphs are indicating fold enrichment with a *P* ≤ 0.05.

Coupling our *in vitro* sporozoite bioassays allowed us to categorize sporozoite genes into three phenotype conditions: (i) quiescent; (ii) invasion-activated; and (iii) early liver-stage (Fig. 2a and Supplementary Data S3). Genes preferentially expressed in salivary glands at RT in insect media without mammalian serum components (BSA) defined the quiescent phenotype. These quiescent-state genes were identified as genes most highly expressed in unperturbed PvSGSs by multiple pairwise comparisons of different microenvironment activators between the key insect-like and mammalian-like variables, including temperature (37 °C vs RT °C, log2FC < −1, *P* (*p* value) ≤ 0.05) (Supplementary Data S4) and insect or mammalian media (with or without BSA, log2FC < −2, *P* ≤ 0.05) (Supplementary Data S5). High transcript abundance implicated gliding motility proteins such as TRAP-like protein (TLP) and others (e.g., ROM1) as representative of the quiescent stage. Invasion-activated genes, which were genes preferentially expressed in the early phase (4 h vs. 0 h, log2FC < −2, *P* ≤ 0.05, Supplementary Data S6) and induced by BSA, included > 100 invasion- and motility-related genes (RPMI +3% BSA, 0 h vs. RPMI +3% BSA, 4 h, 37 °C, log2FC < −2, *P* ≤ 0.05, Supplementary Data S7). Many leading and attempted vaccine candidates are included in the invasion group, such as CelTOS, Apical Membrane Antigen 1 (AMA1) and Thrombospondin-related Adhesive Protein (TRAP). Finally, the early liver-stage is represented by genes with enriched expression at core human body temperature 37 °C (4 h, 37 °C vs. 4 h, RT, log2FC > 2, *P* ≤ 0.05, Supplementary Data S8). The early liver-stage is also the largest group, representing a major transcriptome shift when the motile parasite transitions to the intracellular microenvironment and initiates liver-stage parasite development. As expected for intracellular developmental requirements, several hundred up-regulated genes are related to protein biosynthesis, fatty acid synthesis, and mitochondrial function. To analyze these important parameters in our experimental setup, i.e. elapsed time (0 h and 4 h), host temperature shifts (RT and 37 °C), and host chemical environment change (with and without BSA), we calculated the overall transcriptome shift as indicated by Pearson’s r between different experimental conditions. We used the transcriptomes of freshly-dissected sporozoites (0 h post-dissection) as a baseline of comparison and calculated the similarities between 4 h post-dissection with varying BSA and 37 °C conditions. We found a large overall change of transcriptome induced by temperature, with an r decrease to 0.2, when the parasite is maintained at 37 °C. The most significant transcriptome change occurred when both BSA and 37 °C are combined, with an r decrease of 0.3 to 0.5 (Fig. 2b).

### An insect-like microenvironment improves sporozoite vitality and hepatocyte invasion

To define the most suitable conditions to sustain viability and infectivity of freshly-dissected PvSGSs, pH and time were experimentally assessed using dissection into Schneider’s insect media in comparison to RPMI. For all experiments, sporozoite viability and infectivity were measured using *in vitro* assays of sporozoite invasion of cultured PHHs and quantification of development of liver-stage parasites after 6–8 days post-infection. Collection of freshly dissected PvSGSs into Schneider’s, pH 4 adversely affected sporozoite viability, resulting in no hepatocyte invasion and liver-stage parasite development, whereas Schneider’s, pH 7 resulted in the highest rates of PHH infection and liver-stage parasite development (3-fold higher than RPMI) (Supplementary Fig. S3a). When collected PvSGSs were maintained for 24 hours (at 4 °C or RT), only PvSGSs in Schneider’s, pH 7 or Schneider’s +3% BSA, pH 7 retained the ability to invade hepatocytes showing a ~2-fold increase in liver-stage parasites when the PvSGSs were maintained at RT (Supplementary Fig. 3b). These studies demonstrated that sporozoites-infected salivary glands collected into Schneider’s, pH 7 are most efficient for hepatocyte infection, and these conditions are capable of maintaining sporozoite viability for at least 24 hours. The media Schneider’s, pH7 was thus selected as the insect-like environment for our RNA-seq study and is referred to as “Schneider’s” hereafter.

**Figure 3:**
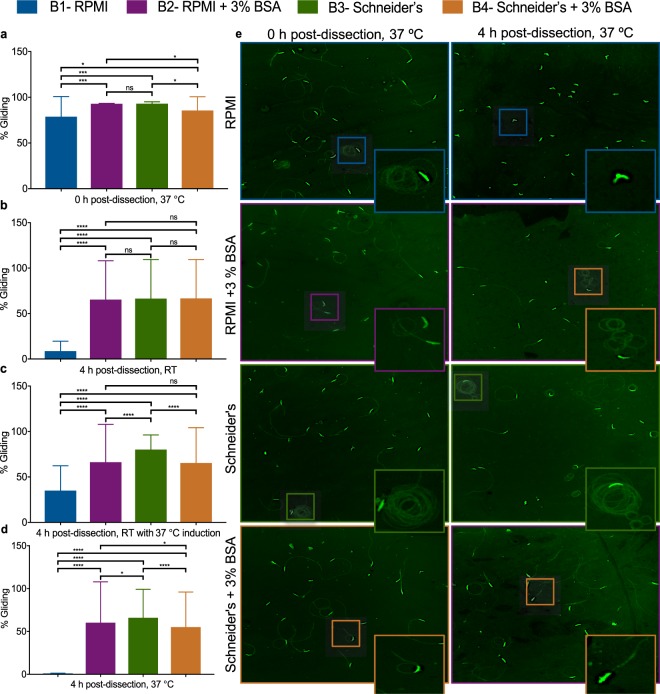
Human body temperature and albumin serves as triggers for sporozoite motility while insect microenvironment maintains sporozoite viability. (**a**) Imaged *P. vivax* salivary gland sporozoites (PvSGSs) exhibit gliding motility in all experimental microenvironments upon rupture from salivary glands during 0 h post-dissection, 37 °C (T0, 37 °C). (**b**) PvSGSs maintained for 4 h post-dissection at room temperature (RT) retain gliding motility in microenvironments induced by buffered mediums containing BSA (RPMI + 3% BSA and Schneider’s + 3% BSA), or in insect-like media, Schneider’s. (**c**) PvSGSs in mammalian-like microenvironment (RPMI) reactivate gliding motility when maintained 4 h post-dissection, RT and subjected to a 30 min induction at 37 °C (**d**) Only PvSGSs maintained in insect-like microenvironment (Schneider’s) show circular motility patterns 4 h post-dissection,37 °C. (**e**) Representative images of PvSGSs gliding motility at 0 h and 4 h post-dissection, 37 °C. A standard sandwich immunofluorescence assay (IFA) based on the monoclonal anti-circumsporozoite protein (CSP) antibody was used to visualize sporozoite motility where manual quantification (from 10 fields of view or 1,000 total PvSGSs) was used to measure sporozoite metrics (percent gliding, motility path). All images were captured at 20x magnification, 0.74 NA. Scale bars (white) represent 10 µM. Graph bars represent mean with s.d for experimental replicates (n = 10) and biological replicate (n = 3) from 10 fields of view or 1,000 PvSGSs. Statistical significance was calculated using a two-way ANOVA with Tukey’s multiple comparisons test to all means where statistical significance values are represented as *P* < 0.05 (*), *P* < 0.001 (***), and *P* < 0.0001 (****). (Blue = RPMI, Purple = RPMI + 3% BSA, Green = Schneider’s, Orange = Schneider’s + 3% BSA).

**Figure 4:**
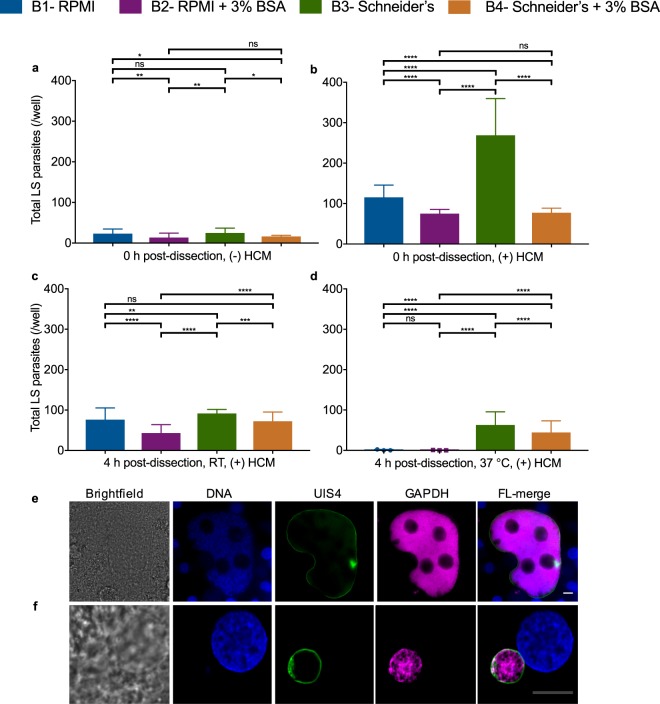
Hepatocyte culture media (HCM) increases *P. vivax* salivary gland sporozoites (PvSGSs) invasion of primary human hepatocytes (PHHs). (**a**) The 0 h post-dissection (−) hepatocyte culture media (HCM) refers to PvSGSs collected, diluted, and inoculated in PHHs using respective experimental media and no addition of HCM where all showed low formation of liver-stage parasites. (**b**) PvSGSs diluted in HCM containing non-heat inactivated serum (0 h post-dissection, (+) HCM) show increased liver-stage parasite development with highest rates in sporozoites dissected into Schneider’s. (**c**) PvSGSs show a decrease in liver-stage parasite development following an incubation at RT (room temperature) for 4 h post-dissection compared to 0 h post-dissection, (+) HCM) where the mammalian media-based microenvironment (RPMI) had the lowest rates. (**d**) PvSGSs maintained for 4 h post-dissection at 37 °C lack liver-stage parasite development in mammalian media-based microenvironments (RPMI and RPMI + 3% BSA) while sporozoites in insect media-based microenvironments (Schneider’s and Schneider’s + 3% BSA) retain PHH infectivity. (**e**) Representative image of a *P. vivax* developing schizont on day 8 post-infection while (**f**) is a representative image of a day 8 *P. vivax* hypnozoite where both are positively stained by anti-UIS4 and anti-GAPDH. All PHH invasion assays were performed with 5.0 × 10^3^ sporozoites per well inoculum with fixation on day 8 post-infection. Graph bars represent means with s.d for experimental replicates (n = 5 or 6) and biological replicates (n = 3). Statistical significance was calculated using a two-way ANOVA with Tukey’s multiple comparisons test to all means where statistical significance values are represented as *P* < 0.05 (*), *P* < 0.005 (**), *P* < 0.001 (***), *P* < 0.0001 (****). Scale bars white represent 10 µm and grey represent 5 µm. (Blue = RPMI, Purple = RPMI + 3% BSA, Green = Schneider’s, Orange = Schneider’s + 3% BSA).

The PvSGSs samples collected for RNA-seq were also simultaneously subjected to bioassays measuring sporozoite motility and invasion of PHHs. We found PvSGSs exposed to Schneider’s insect media showed a prolonged gliding motility phenotype across all time points (>60%) in comparison to RPMI which had a large reduction to <10% gliding after time 0 (Fig. 3a–e and Supplementary Fig. S4). Also, significant differences in infectivity and liver-stage parasite development resulted from exposure to the different microenvironments (Fig. 4a–d). Of highest importance, PvSGSs dissected into Schneider’s with subsequent addition of serum-containing hepatocyte culture media (HCM) at the time of hepatocyte infection produced superior PHH infection rates with complete development of day 8 liver-stage schizonts and hypnozoites. At 0 h post-dissection, PvSGSs collected in Schneider’s were inoculated in PHHs at 5 × 10^3^ sporozoites per well generating an average of 269 ± 91 liver-stage parasites per well of a 384-well plate, which is greater than a 2-fold increase over collection media RPMI of 115 ± 30 liver-stage parasites (Fig. 4b). Similarly, at 4 h post-dissection, 37 °C only PvSGSs in Schneider’s and Schneider’s + BSA mediums successfully infected PHHs (Fig. 4d). Immunofluorescence staining of the parasitophorous vacuole membrane (PVM) biomarker Upregulated in Infective Sporozoites 4 (UIS4) and active glycolysis biomarker Glyceraldehyde 3-Phosphate Dehydrogenase (GAPDH) were used to identify liver-stage parasites and distinguish between developing schizonts and hypnozoites (Fig. 4e–f)^43^. All liver-stage parasites were imaged, classified, and quantified on a high-content imaging system (Operetta, Perkin Elmer) following an established methodology previously described^44^.

### Identification of genes involved in sporozoite quiescent state

To discover the molecular metabolic activities of the sporozoite quiescent state, differentially expressed genes were identified between insect-like sporozoites at 0 h, (Schneider’s, RT) with environmentally activated sporozoites (4 h post-dissection, RPMI + 3% BSA, 37 °C). Transcriptome profiling revealed 197 genes down-regulated upon entry into the human-like environment with the highest annotated differentially expressed genes summarized in Table 1. Of the 197 differentially expressed genes, 74 are unknown with the remaining 123 having annotated sporozoite-associated roles (Supplementary Data S3). Familiar annotated proteins expressed include the cell traversal proteins Gamete Egress and Sporozoite Traversal protein (GEST), Sporozoite Protein Essential for Cell Traversal 1 (SPECT1), CelTOS, and the motility/invasion related proteins Thrombospondin-Related Adhesive Protein (TRAP), TRAP-like Protein (TLP,) Thrombospondin-Related Sporozoite Protein (TRSP), CSP, Secreted Protein with Altered Thrombospondin Repeat domain (SPATR), and profilin (PFN). Notably, our differential-expression analysis revealed genes involved in intracellular calcium signaling cascades, including Calcium-Dependent Protein Kinase (CDPK) 4, centrin (CEN) 2, and CDPK6, indicating proteins are maintained by sporozoites prior to stimulation from the mammalian host (Fig. 2c) (FPKM ≥ 20, log2FC ≥ 1, *P* ≤ 0.05). Further, our results implicate genes with unexplored but hypothesized roles in sporozoites such as the recently characterized proteolytic cascade in *P. falciparum* blood-stage involving Plasmepsin X (PMX), subtilisin 2 (SUB2), and AMA1 used for invasion (FPKM ≥ 20, log2FC ≥ 1, *P* ≤ 0.05)^45^. In total, we identified 74 genes with unknown functions that we predict may have a role in the sporozoite pre-invasive, quiescent state. Among the top most highly expressed were PVP01_0943600, PVP01_0210600, and PVP01_0319000 (FPKM ≥ 20, log2FC ≥ 2, *P* ≤ 0.05) (Supplementary Data S3).

**Table 1:** 30 genes with annotations most highly up-regulated in quiescent *P. vivax* sporozoites.

**Gene ID**	**Product description**	**Gene symbol**	***p*** **-value**
PVP01_1105500	nucleoside diphosphate kinase, putative	NDK	<0.0001
PVP01_1132600	TRAP-like protein, putative	TLP	<0.0001
PVP01_0613800	merozoite TRAP-like protein, putative	MTRAP	<0.0001
PVP01_1226800	nicotinamide/nicotinic acid mononucleotide adenylyltransferase, putative	NMNAT	0.0093
PVP01_1124400	sphingomyelin synthase 2, putative	SMS2	0.0160
PVP01_0607900	phospholipid scramblase, putative		0.0003
PVP01_1258000	gamete egress and sporozoite traversal protein, putative	GEST	0.0143
PVP01_1249700	thioredoxin 1, putative	TRX1	<0.0001
PVP01_0920900	CorA-like Mg2+ transporter protein, putative	MIT1	<0.0001
PVP01_1245400	phosphatidylinositol-4-phosphate 5-kinase, putative	PIP5K	<0.0001
PVP01_0615300	claudin-like apicomplexan microneme protein, putative	CLAMP	<0.0001
PVP01_1224800	apicoplast calcium binding protein 1, putative	ACBP1	<0.0001
PVP01_1436800	thrombospondin-related apical membrane protein, putative	TRAMP	0.0010
PVP01_1321700	CorA-like Mg2+ transporter protein, putative	MIT3	0.0003
PVP01_1212700	iron regulatory protein, putative	IRP	<0.0001
PVP01_0114800	serine/threonine protein kinase, FIKK family	FIKK	<0.0001
PVP01_0943700	alpha/beta hydrolase, putative	alpha/beta hydrolase	<0.0001
PVP01_0928000	tRNA (guanine-N (7)-)-methyltransferase, putative	tRNA (guanine-N (7)-)-methyltransferase	<0.0001
PVP01_0728800	merozoite surface protein 1 paralog	MSP1P	0.0010
PVP01_1223600	protein kinase, putative	PK	<0.0001
PVP01_0313300	calcium-dependent protein kinase 4, putative	CDPK4	0.0014
PVP01_1141700	uroporphyrinogen III decarboxylase, putative	UROD	<0.0001
PVP01_1456100	COPI associated protein, putative	COPI	<0.0001
PVP01_1464100	DNA replication origin binding protein, putative	DIA2	0.0007
PVP01_0112200	plasmepsin X, putative	PMX	<0.0001
PVP01_1322600	phosphoenolpyruvate carboxylase, putative	PEPC	0.0171
PVP01_0303900	6-cysteine protein, putative, pseudogene	6-cys	<0.0001
PVP01_0921000	alpha/beta hydrolase fold domain containing protein, putative	alpha/beta hydrolase	<0.0001
PVP01_0519100	vacuolar protein sorting-associated protein 2, putative	VPS2	0.0002
PVP01_0906300	centrin-4, putative	CEN4	<0.0001

This table represents differentially expressed genes (0 h vs 4 h, 37 °C) with exclusion of unannotated genes.

Refer to Supplemental Data S3 for complete data set.

The function of the *Plasmodium* apicoplast is indispensable for the parasites survival, but the organelle’s function is not fully defined. The Suf family is one of the four known pathways for Fe-S cluster biogenesis and is believed to provide the Fe-S clusters to many proteins functional in the apicoplast, including proteins involved in the isoprenoids biosynthesis pathway. Our analysis indicated an increased abundance of apicoplast genes, suggesting the organelle may have a role in both quiescent sporozoites (SufE) and early liver-stage parasite formation. To validate RNA-seq data, four genes of the Suf family (SufE, SufA, SufS, SufC) and the most conserved protein of the isoprenoid pathway (IspD)^46^ were analyzed by qPCR (n = 2) which confirmed SufE and SufS are actively expressed (Supplementary Fig. S5a). Further, high-resolution fluorescent microscopy of *P. vivax* sporozoites using mouse immune sera raised against proteins SufS and SufE stained positive for SufS in a single sporozoite organelle, similarly to an Acyl Carrier Protein (ACP) control with known localization to the apicoplast. Alternatively, SufE had a diffused staining pattern with localization in multiple sporozoite organelles (Supplementary Fig. S5b). These data indicate the potential importance of this pathway during sporozoite and liver-stages, warranting further investigation.

Lastly, to provide biological insight into processes involved in sporozoite quiescence, we performed an enrichment analysis of the identified differentially expressed genes using respective functional annotation in the Gene Ontology (GO) and KEGG databases (Fig. 2d)^47,48^. In total, 4 cellular-components GO-terms were enriched: cell surface (n = 12), microneme (n = 8), cell periphery (n = 6), and osmiophilic body (n = 3) (*P* ≤ 0.05) (Fig. 2d). Further, >40% of differentially expressed genes were enriched in molecular functions involving catalytic activity, including transferase activity (n = 30), metal ion binding (n = 20), protein kinase (n = 10), oxidoreductase activity (n = 10), lyase activity (n = 6), iron-sulfur cluster binding (n = 4), and anion transmembrane transport activity (n = 2) (*P* ≤ 0.05) (Fig. 2d). Biological process GO-terms including locomotion (n = 7), phosphorylation (n = 12), macromolecule modification (n = 22) were enriched (*P* ≤ 0.05) (Fig. 2d). Moreover, 8 genes were found to participate in carbon-fixation pathways in prokaryotes (ec00720) and the citrate cycle (TCA) (ec00020).

### Quiescent sporozoites are activated by human serum components

The PvSGSs samples from 0 h were used to compare all buffered mediums to identify PvSGS genes triggered by induction to the mammalian-like environment. A total of 16 up-regulated genes were differentially expressed upon BSA activation summarized in Table 2. The results showed 11 annotated genes with the intracellular calcium cascade modulator CDPK1 most highly expressed followed by sporozoite surface protein 3 (SSP3), ADP-ribosylation factor GTPase-activating protein (APH), sporozoite surface protein essential for liver stage development (SPELD), syntaxin (SYN17), ADP-ribosylation factor GTPase-activating protein (ARFGAP), and rho GTPase-activating protein (PVP01_0806900) (FPKM ≥ 20, log2FC ≥ 1, *P* ≤ 0.05). Our bioassays suggest phenotypic associations with these differentially expressed genes, showing a mean of 96% ± 2% PvSGSs gliding in RPMI + 3% BSA compared to 78% ± 22% in RPMI at 0 h post-dissection, 37 °C (*P* < 0.0001) (Fig. 3a,e). Furthermore, BSA activation extends PvSGSs gliding motility at both RT and 37 °C (65% ± 43% and 60% ± 48%). In comparison, virtually no gliding was evident in RPMI (9% ± 11% and 0.5% ± 0.9%); apparent arrest of sporozoite migration and transformation into liver-stage parasites were marked by early PVM formation (Fig. 3b,d,e and Supplementary Fig. S3). Importantly, activation of PvSGSs by the mammalian environment or with addition of BSA impeded sporozoite ability to invade PHHs as shown by a drastic reduction in liver-stage parasite developmental rates (Fig. 4a–d). At 0 h post-dissection, PvSGSs collected in RPMI + 3% BSA or Schneider’s + 3% BSA had an average of 75 ± 10 or 77 ± 11 liver-stage parasites per well, respectively (Fig. 4b).

**Table 2:** Genes with annotations most highly up-regulated in activated *P. vivax* sporozoites.

**Gene ID**	**Product description**	**Gene symbol**	***p*** **-value**
PVP01_0407500	calcium-dependent protein kinase 1, putative	CDPK1	0.0285
PVP01_1032700	conserved Plasmodium protein, unknown function		0.0229
PVP01_0806900	rho GTPase-activating protein, putative		0.0247
PVP01_1427900	sporozoite surface protein 3, putative	SSP3	0.0054
PVP01_0521100	acylated pleckstrin-homology domain-containing protein, putative	APH	0.0084
PVP01_0938800	sporozoite surface protein essential for liver stage development, putative	SPELD	0.0225
PVP01_0414200	syntaxin, Qa-SNARE family, putative	SYN17	0.0001
PVP01_1461700	ADP-ribosylation factor GTPase-activating protein, putative	ARFGAP	0.0118
PVP01_0508000	SPRY domain, putative		0.0118
PVP01_0940400	adrenodoxin reductase, putative		0.0008
PVP01_1236500	conserved Plasmodium protein, unknown function		0.0147
PVP01_1461800	conserved Plasmodium protein, unknown function		0.0104
PVP01_1010500	DnaJ protein, putative		0.0477
PVP01_1423500	calmodulin, putative		0.0074
PVP01_1262000	conserved Plasmodium protein, unknown function		0.0052
PVP01_1460500	conserved Plasmodium protein, unknown function		0.0048

This table represents differentially expressed genes (0 h vs 4 h, 37 °C) with exclusion of unannotated genes.

Refer to Supplemental Data S3 for complete data set.

### Specific human factors induce sporozoite transition into an early liver-stage parasite

After a sporozoite invades the host hepatocyte, formation of a PVM ensues for transition into a highly metabolically-active intracellular liver-stage parasite preparing for rapid replication. To explore the early liver-stage parasite transcriptomic profile, an analysis of differentially expressed genes was performed using our PvSGSs exposed to the mammalian-like environment (4 h post-dissection, 37 °C). We identified 518 differentially expressed genes noting 30 highly abundant annotated differentially expressed genes in Table 3 (Supplementary Data S3). Interestingly, the ookinete surface protein p25 (Pvs25), potentially a false-positive, followed by the Parasitophorous Vacuole protein 1 (PV1) showed the highest differential expression with temperature change and BSA induction. Another intriguing find was the expression of Sortilin, recently characterized in *P. falciparum* asexual blood-stage as a transmembrane protein involved in protein trafficking to rhoptries of the apical complex, suggesting a role in the sporozoites secretory system during and after host hepatocyte invasion^49,50^. Additional genes implicated in initial liver-stage parasite development include Liver Stage Associated Protein 1 (LSAP1), Sporozoite Invasion-Associated Protein 2 (SIAP2) and four PVM-associated genes (PVP01_0504800, PVP01_1271000, PVP01_073480, PVP01_0602100) termed Early-Transcribed Membrane Proteins (ETRAMPs), and UIS4. Activation of sporozoite intracellular calcium signaling cascades in response to the mammalian microenvironment was evident with increased abundance of calmodulin, CDPK7, protein kinase 4 (PK4), and Receptor of Activated Protein C Kinase 1 (RACK1). Our results also support recent reports that heat shock proteins (HSP) are critical for liver-stage parasite development as HSP70, HSP70-2, HSP90, and PVP01_1405400 were amongst the highest differentially expressed genes^51^. In order to rapidly transition between pivotal life-cycle stages, *Plasmodium* uses translational repression to regulate pre-transcribed mRNA stockpiles that encode required proteins. Our analysis of differentially expressed genes revealed high expression of known translational regulators ALBA-1^52^, ALBA-2, and ALBA-4^53^ indicating these genes as potential global regulators of developing liver-stage parasites (FPKM ≥ 20, log2FC ≥ 2, *P* ≤ 0.05).

**Table 3:** 30 genes with annotations most highly up-regulated in *P. vivax* sporozoites transitioning into early liver-stage parasites.

**Gene ID**	**Product description**	**Gene symbol**	***p*** **-value**
PVP01_0616100	ookinete surface protein P25	Pvs25	0.0001
PVP01_0929800	parasitophorous vacuolar protein 1, putative	PV1	0.0070
PVP01_1444300	haloacid dehalogenase-like hydrolase, putative	HAD3	0.0063
PVP01_1255700	E3 SUMO-protein ligase NSE2, putative	NSE2	0.0001
PVP01_0905900	histone 2B, putative	H2B	<0.0001
PVP01_1131700	histone H2A, putative	H2A	<0.0001
PVP01_1130100	cell division cycle protein 48 homologue, putative		0.0025
PVP01_1330600	calmodulin, putative		0.0001
PVP01_1265900	KS1 protein precursor, putative		0.0171
PVP01_1267100	triosephosphate isomerase, putative		0.0206
PVP01_0816000	enolase, putative	ENO	0.0034
PVP01_0815800	tubulin binding cofactor c, putative		0.0310
PVP01_1255200	sortilin, putative		0.0080
PVP01_0713100	major facilitator superfamily-related transporter, putative	MFR4	0.0057
PVP01_1238100	eukaryotic initiation factor 4a, putative	eIF4A	0.0002
PVP01_0905800	histone H4, putative	H4	0.0026
PVP01_0507600	receptor for activated c kinase, putative	RACK1	0.0237
PVP01_0808500	nucleolar protein 5, putative	NOP5	0.0066
PVP01_1208500	DNA/RNA-binding protein Alba 2, putative	ALBA2	0.0004
PVP01_1212100	DNA-directed RNA polymerases I, II, and III subunit RPABC4, putative	RPB12	0.0059
PVP01_0515500	U3 small nucleolar ribonucleoprotein protein IMP4, putative	IMP4	0.0081
PVP01_0811500	proteasome subunit beta type-5, putative		0.0064
PVP01_1302200	high mobility group protein B1, putative	HMGB1	0.0104
PVP01_1429700	ATP-dependent RNA helicase DBP1, putative	DBP1	<0.0001
PVP01_1207600	nucleoside transporter 1	NT1	0.0001
PVP01_1432600	proline–tRNA ligase, putative	PRS	0.0406
PVP01_1022800	stearoyl-CoA desaturase, putative	SCD	0.0001
PVP01_1266000	cytoplasmic tRNA 2-thiolation protein 2, putative	NCS2	0.0431
PVP01_0825400	RING finger protein RNF1, putative		<0.0001
PVP01_0504400	sporozoite invasion-associated protein 2, putative	SIAP2	0.0025

This table represents differentially expressed genes (RPMI, 0 h vs. RPMI 4 h, 37 °C) with exclusion of unannotated genes.

Refer to Supplemental Data S3 for complete data set.

A GO enrichment analysis indicated differentially expressed genes in the sporozoite transition into early liver-stage parasites are enriched in biological processes including translation (n = 74), ribosome biogenesis (n = 14), cellular macromolecular complex assembly (n = 14), and chromosome organization (n = 6) (*P* ≤ 0.05) (Fig. 2e). In terms of cellular components, a majority of genes are associated with the ribosome (n = 64), nucleus (n = 46), and cytosol (n = 56) (*P* ≤ 0.05) (Fig. 2e). Significantly enriched molecular functions GO-terms included nucleic acid-binding (n = 93), RNA-binding (n = 46), and structural constituent of ribosome (n = 62) (*P* ≤ 0.05) (Fig. 2e). Of the KEGG pathways, 4 genes were identified in the pentose phosphate pathway (ec00030), as well as 4 genes involved in carbon fixation in photosynthetic organisms (ec00710).

### Characterization of the *Anopheles dirus* salivary gland transcriptome

**Figure 5:**
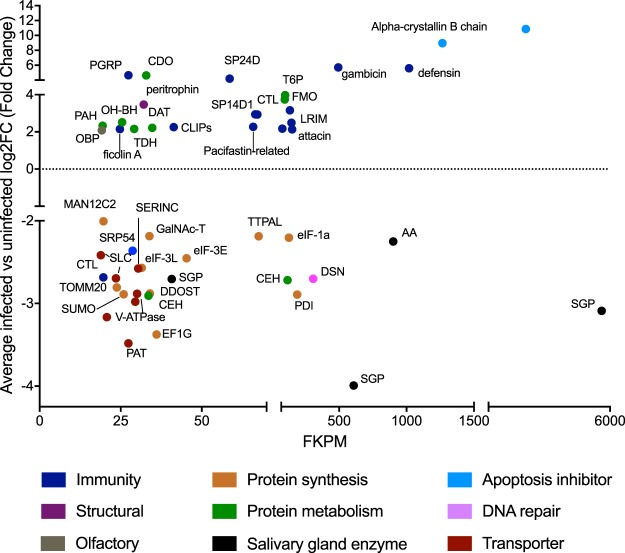
Characterization of the *Anopheles dirus* salivary gland transcriptome. Differential expression analysis revealed up-regulation of 23 genes with known functions in mosquito immune factors, metabolism, apoptosis inhibition, structural, and olfactory proteins along with 28 genes down-regulated with functions as solute transporters, protein synthesis, salivary gland enzymes, and DNA repair (averaged infected vs average uninfected, FPKM ≥ 20, *P* ≤ 0.05).

Experimental PvSGS-infected *An. dirus* samples and uninfected *An. dirus* salivary gland controls were analyzed for expression of genes on day 14 (post-blood meal). Transcriptome analysis showed similar expression levels for salivary gland proteins in all samples including salivary gland protein 6 (SGP6), D7 long-form salivary protein, salivary gland protein 1-like, and TRIO salivary gland protein (Supplementary Data S9). The top expressed known gene encoded was anopheline antiplatelet protein (AAPP), which is secreted by blood-feeding females to block platelet adhesion to collagen and prevent aggregation^54^. To explore the transcriptome of PvSGS-infected *An. dirus* salivary glands, differentially expressed genes were compared between infected and uninfected salivary glands. The results showed 71 genes up-regulated and 47 genes down-regulated differentially expressed genes, but only 43% of these differentially expressed genes have a known function (uninfected vs infected, log2FC ≥ 2 or ≤−2, *P* ≤ 0.05) (Fig. 5, Table 4, Supplementary Data S10). The top three genes with significantly excess abundance were uncharacterized (ADIR008862, ADIR014872, and ADIR010223) while the most highly-expressed known genes (ADIR007940 and ADIR007941) are members of the HSP20/alpha-crystallin family which have not been previously reported. As expected, results showed high induction of immune response-related genes, such as the anti-microbial proteins (AMPs): gambicin, defensin, cecropin, attacin, and leucine-rich immune protein (LRIM1) (Fig. 5)^55^. Further assignment of biological relevance using Gene Ontology (GO) analysis revealed differences in gene expression of biological processes where infected salivary glands showed an increase of protein catabolism, apoptosis inhibition, structural composition, and olfaction. On the other hand, down-regulation was observed in genes relating to proton and amino acid transport, protein synthesis, and DNA repair (Fig. 5, Table 4)^56^. Interestingly, enrichment in gene expression for uninfected mosquitoes showed increased expression of three genes (ADIR014650, ADIR003224, and ADIR004887) annotated as salivary gland proteins with significantly lower levels of expression in PvSGS-infected salivary gland samples (Supplementary Data S10).

**Table 4:** Genes with annotations most highly up-regulated and down-regulated in *P. vivax* infected and uninfected *Anopheles dirus* mosquitoes.

**Gene ID**	**Product description**	**Gene symbol**	**Assigned group**	***p*** **-value**
**Up-regulated:**				
ADIR00790	alpha-crystallin B chain	alpha-crystallin B chain	Apoptosis inhibitor	<0.0001
ADIR007941	unknown function/alpha-crystallin B chain	alpha-crystallin B chain		0.0001
ADIR010798	defensin anti-microbial peptide	defensin	Immunity	<0.0001
ADIR006825	gambicin anti-microbial peptide	gambicin		<0.0001
ADIR014587	attacin anti-microbial peptide	attacin		0.0016
ADIR003751	C-type lectin	CTL		0.0004
ADIR007855	leucine-rich immune protein (Long)	LRIM		0.0004
ADIR008083	leucine-rich immune protein (Long)	LRIM		0.0026
ADIR003752	C-Type Lectin	CTL		0.0001
ADIR005185	SP14D1	SP14D1		<0.0001
ADIR007695	serine protease SP24D	SP24D		0.0207
ADIR002798	CLIP-domain serine protease	CLIPs		<0.0001
ADIR000960	peptidoglycan-recognition protein	PGRP		0.0001
ADIR007771	ficolin A	ficolin A		0.0016
ADIR006371	trehalose 6-phosphate phosphatase	T6P	Protein Catabolism	0.0001
ADIR011634	flavin-containing monooxygenase	FMO		<0.0001
ADIR008936	diamine acetyltransferase	DAT		<0.0001
ADIR003836	cysteine dioxygenase	CDO		<0.0001
ADIR008493	threonine dehydratase	TDH		0.0010
ADIR001408	4a-hydroxytetrahydrobiopterin dehydratase	OH-BH		0.0379
ADIR007539	phenylalanine-4-hydroxylase	PAH		0.0023
ADIR003591	peritrophin	peritrophin	Structural	0.0152
ADIR010042	odorant binding protein	OBP	Olfaction	0.0168
ADIR002153	pacifastin-related peptide		Unknown	0.0025
**Down-regulated:**				
ADIR014650	salivary gland protein	SGP	Salivary gland enzyme	0.0062
ADIR007445	alpha-amylase	AA		0.0002
ADIR003224	salivary gland protein	SGP		0.0002
ADIR004887	23.4 kDa salivary protein	SGP		0.0215
ADIR001653	duplex-specific nuclease	DSN	DNA repair	<0.0001
ADIR000534	protein disulfide-isomerase	PDI	Protein synthesis	0.0431
ADIR003326	translation initiation factor 1 A	eIF-1a		0.0411
ADIR002569	alpha-tocopherol transfer protein-like protein	TTPAL		0.0311
ADIR004227	eukaryotic translation initiation factor 3 subunit E	eIF-3E		0.0215
ADIR001129	elongation factor 1-gamma	EF1G		0.0116
ADIR004002	dolichyl-diphosphooligosaccharide–protein glycosyltransferase subunit 1	DDOST		0.0122
ADIR009737	UDP-N-acetyl-alpha-D-galactosamine:polypeptide N-acetylgalactosaminyltransferase	GalNAc-T		0.0112
ADIR003797	eukaryotic translation initiation factor 3 subunit L	eIF-3L		0.0011
ADIR001338	small ubiquitin-related modifier	SUMO		0.0296
ADIR000499	translocase of outer mitochondrial membrane 20 homolog	TOMM20		0.0054
ADIR009264	alpha-12C2-Mannosidase	MAN12C2		0.0362
ADIR009439	carboxylic ester hydrolase	CEH	Protein catabolism	0.0019
ADIR010119	carboxylic ester hydrolase	CEH		<0.0001
ADIR010296	serine incorporator	SERINC	Transporter	0.0109
ADIR009494	V-type proton ATPase proteolipid subunit	V-ATPase		0.0165
ADIR006128	+V-type H+-transporting ATPase 21kDa proteolipid subunit	V-ATPase		0.0107
ADIR004742	proton-coupled amino acid transporter	PAT		0.0007
ADIR006703	sodium-independent sulfate anion transporter	SLC		0.0007
ADIR004547	V-type H+-transporting ATPase subunit E	V-ATPase		0.0148
ADIR010593	V-type proton ATPase catalytic subunit A	V-ATPase		0.0245
ADIR000034	solute carrier family 15 member	SLC15		0.0196
ADIR008886	signal recognition particle subunit SRP54	SRP54	Immunity	0.0046
ADIR007705	C-type lectin	CTL		0.0161

The table represents differentially expressed genes of infected vs. uninfected salivary glands with exclusion of unannotated genes.

Refer to Supplemental Data S9 and S10 for complete data sets.

## Discussion

Transmission of *Plasmodium* sporozoites from mosquito to human involves a transition between two highly distinctive environments, insect to mammal, requiring rapid adjustment for parasite survival. Inside the mosquito’s salivary gland, the sporozoite exists in a quiescent state primed for introduction to the mammalian host but not yet activated. Upon inoculation, sporozoites must immediately react to changes in their microenvironment, especially temperature and serum components, to initiate gliding motility and migration to the liver. In conjunction with this activation, changes in gene expression reflect future developmental requirements for sporozoite cell traversal and then parasite development within hepatocytes. In this study, we used microenvironments with extrinsic stimuli to mimic *in vitro* infection process to define key relationships with distinct biological functions. Most notable, post-dissection preservation of quiescence by placing sporozoites in an insect-like microenvironment and delaying strategic activation until inoculating hepatocyte cultures prevented untimely activation of invasion-associated genes to dramatically increase invasion rates into primary human hepatocytes. Additional studies will be needed to determine the specific components that modulate sporozoite activation.

Our experimental method allowed us to examine factors of the different biotic microenvironments in multiple pair-wise comparisons (insect vs mammal, BSA vs no BSA), time (0 h vs 4 h), and temperature (RT vs 37 °C) where differential expression revealed 3 phenotypic states (i) quiescent; (ii) invasion-activated; and (iii) early liver-stage. We found that across different *Plasmodium* species the sporozoite transcriptome have strong similarities with a conserved set of highly expressed genes such as CelTOS, CSP, and TRAP (Fig. 1b,c). However, this study is the first to differentiate between activated vs non-activated sporozoites in relation to key host physiological triggers accompanied with complete transcriptomic analysis (Fig. 2) and quantitative phenotypic assessment (Figs 3, 4). Previous studies in rodent malaria’s and *P. falciparum* have under-explored the host physiological effects on sporozoites, required laborious purification, and are unable to link expression with *in vitro* phenotypes^39,40,42^. Within the last decade, several blood-stage *P. vivax* sequencing projects in genomics^57,58^, transcriptomics^38,59,60^, and proteomics^61,62^ have advanced our insight of parasite biology^63^, however, *P. vivax* sporozoite biology is underrepresented with only one recent publication on the *P. vivax* sporozoite proteome^63,64^. Our analysis provides more understanding of transitional host effects as the first published *P. vivax* sporozoite RNA-seq study and addresses how to improve *in vitro* infectivity and intracellular development of primary human hepatocytes.

The RNA-seq analysis revealed 197 genes expressed by quiescent PvSGSs maintained in an insect-like environment including genes associated in motility (TLP, TRAP, profilin, CLAMP), cell traversal (GEST, CelTOS, SPECT1), and host invasion (CSP, SIAP1, SPATR). Further, our analysis suggests genes with roles in blood-stage motility and invasion such as Plasmepsin X45 and merozoite TRAP-like protein (MTRAP)^65,66^ to potentially have uncharacterized functions in sporozoites or early liver-stage parasites. In agreement with the RNA-seq results, our bioassays revealed the most notable finding that PvSGSs collected in Schneider’s insect media remain quiescent until activation by exposure to hepatocyte culture media (Fig. 3a–b). Strategic activation by serum-containing media just before PHH inoculation increases (>2-fold) the hepatocyte invasion rates in comparison to the mammalian-based media, RPMI. Previous studies have demonstrated that sporozoites are activated by serum albumin^7,8^, other small ligands^67,68^, or temperature^9^ to cause an intracellular calcium flux followed by exocytosis^69^. In agreement, our results demonstrate increased expression of 16 genes including calmodulin, CDPK1, SSP3, SPELD, and regulatory proteins when sporozoites are activated by BSA. CDPK1 orchestrates many processes required for invasion of host cells, including microneme release and activation of actin-myosin motor in PvSGSs^70^, while SSP3 and SPELD are sporozoite surface proteins with functions in motility^71^ and liver-stage parasite maturation^72^. Based on these results it is evident that premature activation of PvSGSs in mammalian-based mediums mistimes the just-in-time cascade of events leading to reduced sporozoite viability and infectivity.

The transcriptome shift from PvSGSs into early liver-stage parasites, mimicked experimentally by exposure to BSA and human body temperature, identified 517 genes with different levels of expression in the transition. Our GO and KEGG enrichment analysis associated the genes with translation and DNA replication with implications in glycolysis and pentose phosphate pathways. As expected, the differentially expressed genes included many liver-stage-associated genes previously determined to be essential for establishment and development (PV1, ETRAMPs, LSAP1)^73^. Much speculation exists of what genes are present in early liver-stage parasite development where our results more conclusively reveal through the up-regulation of genes involved in the secretory pathway, such as Sortilin, and regulatory processes of gene expression, such as translational repressors ALBA 1, 2, and 4. Furthermore, we attempted to discern the role of the *Plasmodium* apicoplast in sporozoites as the organelle is vital for the parasites survival due to the prokaryotic type metabolic pathways functional within. The importance of Fe-S cluster biogenesis has been deemed essential for the apicoplast maintenance in the erythrocytic stages of *P. falciparu*m^74,75^ and in oocyst development of *P. berghei*^76^ but yet to be studied in the pre-erythrocytic stages. Our RNA-seq analysis and qRT-PCR determines that initial components of the Suf pathway (Suf S and E) are actively up-regulated in infectious sporozoites and early liver-stage parasites where diffused immunofluorescence staining of SufE alludes to the possibility of multiple roles for the protein as previously reported in *Arabidopsis thaliana*^77^. Lastly, our analysis helps prioritize development of new vaccine candidates, such as SIAP2, by its link to hepatocyte invasion and subsequent development^9,78,79^, and new drug targets in signaling cascade, such as calmodulin, CPDK7, PK, and RACK1.

We offer the first RNA-seq analysis of uninfected and *Plasmodium*-infected *An. dirus*, a major SE Asia vector for malaria. While the mosquito salivary gland main purpose is to produce saliva and digestive enzymes to breakdown ingested nutrients, it must tolerate the presence of PvSGS for extended periods in order for transmission to be successful. Our study indicates the presence of the parasite has a profound effect on the *An. dirus* with induction of innate immune responses genes to *P. vivax* infections similar to other studies^55,80–85^; however, we identified many genes not yet associated with *Plasmodium* infections, including the odorant binding protein (OBP) ADIR010042, the serine protease (SP24D) ADIR007695, and the small heat shock associated proteins (alpha-crystallin B chain) ADIR007940 and ADIR007941. Heat shock proteins are essential to mosquito survival and induced by biotic factors such as temperature or colonized parasite^86–88^. Presumably, higher expression in *P. vivax*-infected mosquitoes may contribute to the sporozoites need to mediate stress and thermotolerance when transitioning between hosts during multiple mosquito blood meals. Further, HSP20 has specifically been implemented in regulation of sporozoite adhesion and locomotion^89^. Additionally, we found down-regulation of three salivary gland proteins (ADIR014650, ADIR003224, and ADIR004887) in *P. vivax*-infected salivary glands previously unreported. The parasite manipulation of salivary gland proteins may be a strategy used to bypass the activation of the host innate immune response and avoid detection. Overall, our identification of novel mosquito biomarkers can increase understanding of transmission, improve metrics for measuring transmission, and act as targets for transmission blocking.

Research of *P. vivax* usually lags significantly behind that of *P. falciparum* primarily due to the inability to continuously culture *P. vivax* in the laboratory. However, our research in this study with *P. vivax* sporozoites provide innovative changes to broadly enhance malaria sporozoite research. Our experimental analyses of *P. vivax* sporozoites revealed that the microenvironmental changes associated with transition from mosquito into human host trigger distinct processes for initial activation, hepatocyte infection, and liver-stage parasite development. Importantly, distinct calcium-dependent protein kinase signaling pathways associated with these phases of infection represent attractive new drug targets that might be capable of halting migrating sporozoites before they can infect and hide within the liver. Additionally, we offer the first study on mosquito salivary gland gene and proteins involved in *P. vivax* infection and transmission. Altogether our study represents an important advance in understanding mosquito transmission and the key processes regulating sporozoite infectivity of *P. vivax*.

## Methods

### Ethical statement

The Human Subjects protocols for this study was approved by the Institutional Ethics Committee of the Thai Ministry of Public Health and the Human Subjects Research Review Board of the U.S. Army (WRAIR#1949). The protocols conformed to the Helsinki Declaration on ethical principles for medical research involving human subjects (version 2002) and informed written consent was obtained for all volunteers.

### Mosquito colonization, rearing and *P. vivax* infection

For colonization, 3–5-day old adult *An. dirus* was provided blood meals preserved using citrate phosphate dextrose adenine (CPDA-1) via an artificial membrane feeder containing human blood (Interstate Blood Bank, Inc., Memphis, TN), as previously described^90^. After blood-feeding, females were manually mated, eggs obtained, and larvae and adults maintained as described earlier^91^. The blood-fed adults were maintained in the AFRIMS insectary at 25 °C ± 1 °C and 80% relative humidity until transport to the Mae Sod Malaria Clinic, Mae Sod District (Thailand Ministry of Public Health. A 5% solution (v/v) of commercially produced multivitamin syrup (MULTILIM, Atlantic Pharmaceutical Co., Bangkok, Thailand) that included 5% sucrose (LIN, TTR Group, Col, Uthai Thani, Thailand) was as a food source and changed daily^92^.

The *P. vivax* -infected blood was obtained from Thai patients who reported to medical clinics along the Thai–Myanmar border with signs and symptoms of malaria infection and confirmation of vivax malaria parasites in the blood. Thick and thin blood smears were prepared and then examined microscopically for *P. vivax* parasites and gametocyte densities calculated per 200 white blood cells. In total, 20 ml of venous blood was collected in a heparinized tube and maintained at a constant temperature of 37 °C using a water bath circulation system. After starvation for 8 h, 5–7-day old *An. dirus* females were allowed to blood-feed for 30 min using an artificial membrane feeder. Unfed and partially fed mosquitoes were removed, counted, and discarded in accordance with standard protocols. Engorged mosquitoes were then secured in a container and transported to the AFRIMS insectary where they remained until use at AFRIMS or shipment to USF (Tampa, FL). The adults were maintained at temperature and humidity as described earlier and provided with 10% multivitamin sugar solution that was changed daily to reduce fungal and bacteria growth. *P. vivax* infected *An. dirus* mosquitoes were shipped to USF for experiments RNA-seq experiments (n = 1) and preliminary experimental optimization (Supplementary Fig. S3).

### Preparation of sporozoite dissection collection media

The experimental design included exposure of fresh sporozoites to a mammalian-based media (RPMI), an insect-based media (Schneider’s), and an addition of BSA to media at a 3% w/v concentration mimicking the level in human plasma. RPMI used was pre-made with addition of 50 mg ml^−1^ hypoxanthine, 25 mM HEPES, and L-glutamine (Cat. No. CUS-0645, KD medical, Columbia, MD, U.S.A). Schneider’s powder without sodium bicarbonate or calcium chloride (Cat. No. S9895, Sigma-Aldrich, St. Louis, MO, U.S.A.) was reconstituted following manufacture’s protocol in cell culture water (Corning, New York, NY, U.S.A.) with gently stirring until solution was clear and then pH altered near neutral (7.0–7.3) using 1 M NaOH. Complete list of media components is in Supplementary Table S1. The microenvironments were termed as the following: B1 = RPMI, B2 = RPMI + 3% BSA, B3 = Schneider’s, B4 = Schneider’s + 3% BSA. Time points were termed as the following: 0 h refers to time after dissection was finished and sporozoites were taken off ice (4 °C) where 4 h refers to time after dissection was finished and sporozoites are incubated at respective temperatures. Temperature differences at 4 h post-dissection occurred at either room temperature (RT) or 37 °C, 5% CO_2_. The 4 h RT with 37 °C induction refers to a 30 min incubation (the amount of time to run the gliding assay) of 4 h RT samples with 37 °C, 5% CO_2_ incubation.

### Dissection of *P. vivax* salivary gland sporozoites

For all experiments, salivary glands were dissected from *An. dirus* mosquitoes 14–16 days post–infectious blood meal. Mosquitoes were immobilized by cold incubation (−20 °C, 5 min), transferred to ice (4 °C), and individually washed in a 3-step wash: 70% ethanol, 0.5 mg ml^−1^ penicillin-streptomycin and 1 mg ml^−1^ of neomycin (PSN), and 2.5 µg ml^−1^ Fungizone^TM^ diluted in 1× Dulbecco’s Phosphate Buffered Solution (DPBS). Intact, clean salivary glands were collected into 100 μl of collection media with dissection time under an hour. Salivary glands were centrifuged (1600 G, 3 min) with gland disruption using plastic pestle and manually pipetting then counted using a hemocytometer. A total of 200,000–2,000,000 sporozoites were collected per condition for RNA-seq.

### Sporozoite gliding motility assay

The gliding motility assay used was described in a previous study^34^. Briefly, 20,000–40,000 sporozoites were allowed to glide for 30 min in respective media at either room temperature (23 °C) or 37 °C on a glass coverslip coated with 10 µg ml^−1^ of *P. vivax* anti-CSP monoclonal antibodies (mAB) 2F2 (subtype VK210) or 2E10.E11 (subtype VK247) (BEI Resources, NIAID, NIH)^93^. Subsequently, sporozoites were fixed with 4% paraformaldehyde (PFA) for 10 minutes and blocked with 1% bovine serum albumin (BSA) in 1× phosphate buffered saline (PBS) overnight at 4 °C. Sporozoites were stained with 10 µg ml^−1^ of mAB as primary antibody for 1 hour at room temperature followed by staining with 10 µg ml^−1^ secondary goat anti-mouse Alexa Fluor® 488 conjugate (2 µg ml^−1^, 1:1,000-fold dilution; Molecular Probes, ThermoFisher, Waltham, MA, U.S.A.). The coverslip was washed thrice to remove unbound antibodies and mounted inverted on a drop of Fluoromont G. Illuminated anti-CSP trails were quantified and imaged by fluorescence microscopy (20x, 1.4 NA or 100×, 1.4 NA, DeltaVision Core system, GE Healthcare Life Sciences, Piscataway Township, NJ, U.S.A.) where positive gliding was defined by a trail ≥ 3 μm. Percent gliding was calculated by total gliding sporozoites divided by total sporozoites (gliding and non-gliding) for 10 fields of view (20x, 1.4 NA) or for a total of 1,000 sporozoites.

### Infection of primary human hepatocytes with *P. vivax* sporozoites

Cryopreserved primary human hepatocytes (Cat. No. M00995-P and F00995-P, Bioreclamation IVT, Inc, Baltimore, MD, U.S.A.) were previously evaluated for *P. vivax* invasion where donor lots NLX (Supplementary Fig. S3) or PDC (Fig. 4) were used for PHH invasion experiments. In brief, selected wells of a commercial 384-well plate (Cat No. 781091, Greiner, Monroe, NC, U.S.A.) were coated with rat tail collagen I (Corning, New York, NY, U.S.A) diluted to a final concentration of 5 µg collagen per cm^2^ in 0.02 M acetic acid and incubated at 37 °C overnight to ensure adsorption. Next day, wells were washed twice with 1× DPBS and once with InVitroGro^TM^ CP Medium (Bioreclamation IVT, Inc, Baltimore, MD, U.S.A.) referred to as hepatocyte culture media (HCM). Cryopreserved PHH were thawed following manufacturer’s instructions with post–thaw viability measured by trypan blue exclusion with > 5 million viable cells per vial and diluted in HCM supplemented with a final concentration of 50 µg ml^−1^ penicillin-streptomycin, 100 µg ml^−1^ of neomycin, and 10 µg ml^−1^ of gentamicin (HCM^+^) to a concentration of 900 cells per μl. The cell mixture was seeded as a confluent monolayer with a total of 18,000 cells per well in a 384-well plate then incubated at 37 °C, 5% CO2. Following 2 days post–seed, each well received a complete media change (40–50 µl) with HCM^+^ then ready for sporozoite infection.

After dissection, sporozoites were counted using a hemocytometer and diluted accordingly in experimental medium (Fig. 4a) or HCM^+^ (Fig. 4b–d), respectively. After dilution, 5.0 × 10^3^ (Fig. 4a–d) or 1.8 × 10^4^ (Supplementary Fig. S3a,b) sporozoites from each condition were added to hepatocyte-seeded wells with experimental replicates (n = 5 or 6), the plate centrifuged (200 G, 5 min), and allowed to invade for 24 hours at 37 °C, 5% CO2 before washing with HCM^+^ (40–50 µl). Media was changed every 2 days using HCM^+^ until fixation at 6–8 days post-infection. To fix cells, 4% PFA was incubated for 10 min at room temperature and washed twice with 1× DPBS then stored at 4 °C until temperature-controlled shipment to USF.

### Microscopy and image analysis of *P. vivax* liver-stage parasites

In brief, a standard immunofluorescence assay (IFA) protocol was used to stain and quantify *P. vivax* liver-stage parasites. Wells were incubated in blocking buffer (0.03% TritonX-100, 1% (w/v) BSA in 1× DPBS) with rabbit anti-UIS-4 polyclonal antibody or mouse anti-ACP antibody at 1:1,000-fold dilution (kindly provided by Center for Infectious Disease Research, WA, U.S.A) and mouse anti-GAPDH monoclonal antibody at 1:50,000-fold dilution (The European Malaria Reagent Repository, Cat. No. 7.2) overnight at 4 °C^43,94^. After, wells were washed thrice with 1× DPBS and incubated for 1 hour at room temperature with secondary goat anti-mouse or anti-rabbit Alexa Fluor® 488 or 568 conjugate at 1:1,000-fold dilution (2 µg ml^−1^; Cat. No. A11001, A11034, A11004, A11011, Molecular Probes, ThermoFisher, Waltham, MA, U.S.A.) antibody and Hoechst at 1:1,000-fold dilution (10 µg ml^−1^) then washed thrice and filled with 1× DPBS until imaging and for long-term storage. Liver-stage parasite imaging, identification, and quantification was performed on the Operetta Imaging System with Harmony software 4.1 (20x, 0.4 NA; Perkin Elmer, Waltham, MA, U.S.A.) following previously described protocol^44^. Each individual well of a 384-well plate had 35 fields of view collected, capturing anti-UIS4 signal using FITC channel and hepatocyte nuclei using DAPI channel. Predetermined image analysis parameters were set to collect the total liver-stage parasite population. Large, developing liver-stage schizonts and small, dormant hypnozoites were distinguished based upon fluorescent intensity, area, and roundness. Statistical analysis of PHH invasion assays were performed using Prism 7 (Graphpad, La Jolla, CA, U.S.A.).

High-resolution IFA images of the *P. vivax* liver-stage parasites were captured with a 100x oil objective, 1.4 NA on a DeltaVision Core system (GE Healthcare Life Sciences, Piscataway Township, NJ, U.S.A.). The same IFA protocol was followed as described above. Mouse immune sera samples collected from mice raised against protein to SufE and SufS were diluted in blocking buffer at 1:50 or 1:200 (kindly provided by V.S. and G.S.S., Z.P. and S.G). All high-resolution images were deconvoluted using the softWoRx® image analysis package (GE Healthcare Life Sciences, Piscataway Township, NJ, U.S.A.) and merged z-stacks with added scale bars were processed in (ImageJ)^95^. Images captured with secondary antibody Alexa Fluor® 568 were re-colored to magenta using FIJI in order to meet publication image standards.

### Library preparation and RNA-sequencing of sporozoites and uninfected mosquitoes

The *P. vivax* sporozoite samples and uninfected salivary glands were extracted and purified from TRIzol using Direct-zolTM RNA miniprep kit (Zymo Research, Irvine, CA, U.S.A.). Sample protein concentration and quality were then measured on a Nanodrop (ThermoFisher, Waltham, MA, U.S.A.). 0.5 μg–1.0 μg total RNA of each sample was used for library preparation. Libraries were prepared by using TruSeq Stranded mRNA Preparation Kit v2 (Illumina, San Diego, CA, U.S.A.) following manufacturer’s recommendations. Library quantification was conducted by qPCR and TapeStation (Agilent Technologies, Santa Clara, CA, U.S.A.) measurements. Libraries were subsequently pooled and sequenced across 4 or 12 lanes of a MiSeq at a read length of 100 bp paired end using 300 cycle V2 MiSeq reagent kit (Illumina, San Diego, CA, U.S.A.).

### Mapping, data processing, and transcriptome profiles

The sequencing raw reads from each sporozoite sample were aligned to the *P. vivax* reference genome P01^58^ of PlasmoDB version 34^96^. The sequencing raw reads from each infected and uninfected *An. dirus* salivary gland samples were aligned to the *An. dirus* WRAIR2 reference genome of VectorBase release VB-2018-02 (AdirW1.7)^56^. A maximum of one mismatch per read was allowed. The mapped reads from TopHat^97^ were used to assemble known transcripts from the reference and their abundances were estimated using Cufflinks^98^. The expression level of each gene was normalized as Fragments Per Kilobase of exon per Million (FPKM) mapped reads for each condition. All the RNA sequencing raw reads have been deposited into NCBI’s Gene Expression Omnibus which are accessible through GEO Series accession number GSE117538.

### Differential gene expression analysis

The difference of gene expression levels across the different time points and buffered media conditions were determined from fragment counts using EdgeR^99^ in R software^100^. The aligned read pairs from sorted bam files were counted against *P. vivax P01* (PlasmoDB v34) and *An. dirus* WRAIR2 (AdirW1.7) gene annotations using featureCounts application of the Subread suite^101^, defining features at the gene-level. TMM (trimmed mean of M-values) normalization^100^ of the counts was performed across the five runs and three biological replicates to eliminate compositional biases between the samples. For each pairwise comparison (Supplementary Dataset S4–S9) performed, a gene was inferred differentially expressed if the FPKM ≥ 20, log2FC of the mean FPKM across biological replicates is greater 2 or less than −2, *P* ≤ 0.05 and FDR (False Detection Rate) < 0.1.

### Gene Ontology (GO) term enrichment analysis

Predetermined gene lists from each pairwise differential gene expression comparison were used to test the enrichment of specific GO term in each group. The GO enrichment analysis of the molecular function, cellular component, and biological processes were conducted using the Gene Ontology (GO) tool on PlasmoDB^96^ (www.plasmodb.org) and analyzed using a *P* of ≤0.05 cutoff. REVIGO program^102^ was used to reduce the redundancy in GO terms. GO terms with a frequency ≥20% were reported and fold enrichment was used to generate figures.

### Gene expression comparison of human and mosquito stages of *P. vivax*

Gene expression comparison between recently published *P. vivax* blood-stage^38^ and our current sporozoite stage transcriptomic data, to understand potential underlying variations and identify sporozoite specific genes (Supplementary Dataset S3, Supplementary Fig. S2). Raw reads from sporozoite samples are re-aligned^98^ to *P. vivax* reference genome Sal1 of PlasmoDB version 12 to be consistent with blood-stage RNA-seq data. Peak gene expression across the sporozoite (buffered media and time point) and blood (time point) samples are respectively profiled along with peak expression point, to classify the genes and rank genes based on sporozoite stage expression.

### Quantitative real time RT-PCR (qRT-PCR)

Total RNA was extracted from samples preserved in Trizol as described above. After giving DNase I (Invitrogen, Carlsbad, CA, U.S.A.) treatment, the total RNA (about 50–100 ng) was subjected to first-strand cDNA synthesis using the Superscript II Reverse Transcriptase kit (Invitrogen, Carlsbad, CA, U.S.A.) and gene-specific primers following manufacturer’s protocol. The resulting cDNA was diluted 1:5 with nuclease-free water. For real time analysis, the primers were designed based on mRNA of desired genes available from PlasmoDB (Supplementary Table S2) using freely available software GeneRunner and cross verified using Primer Blast (NCBI). Real-time PCR analysis was performed on Agilent Technologies Stratagene Mx3005P System using the Brilliant II SYBR Green QPCR Master Mix with low ROX (Agilent Technologies, Santa Clara, CA, U.S.A.). Briefly, the PCR reaction consisted of 12.5 µl of Brilliant II SYBR Green QPCR Master Mix, 10 µmole of forward and reverse primers and 2 µl of diluted cDNA in a total volume of 25 µl. PCR cycling conditions were performed using the default conditions of the Mx3005P Software: 95 °C, 10 minutes followed by 40 cycles of 94 °C, 30 seconds; 55 °C, 1 minute; and 72 °C, 30 seconds. The housekeeping *P. vivax* gene, seryl-tRNA- synthetase, known to be transcribed stably throughout different erythrocytic stages was used as an endogenous control (normalizer) for every run. Two buffered media compositions (RPMI + 3% BSA, Schneider’s) were used from 2 biological samples and run in triplicates for the following timepoints, 0 h post-dissection and 4 h post-dissection, 37 °C. For data analysis, the normalized quantity of each target gene was expressed as the ratio of the relative amount of target gene over the quantity of the housekeeping gene. Then, log2FC was used calculated from qPCR data (Schneider’s, 0 h post-dissection vs. RPMI + 3% BSA, 4 h post-dissection, 37 °C) with results compared to the RNA-seq generated FPKM (Supplementary Fig. S5a).

### Statistical analysis

All graph bars are represented by means with standard deviation (s.d.). Significance of mean was assessed by two-way ANOVA with Tukey’s multiple comparisons (Figs 3 and 4) When standard deviation is reported, n represents biological replicates and experimental replicates per biological replicate. When *p* value is reported, *P* is used for representation.

### Disclaimer

Material has been reviewed by the Walter Reed Army Institute of Research. There is no objection to its presentation and/or publication. The opinions or assertions contained herein are the private views of the authors, and are not to be construed as official, or as reflecting true views of the Department of the Army or the Department of Defense. The investigators have adhered to the policies for protection of human subjects as prescribed in AR 70–25.

## Electronic supplementary material

Supplementary Information

Supplementary Dataset 1

Supplementary Dataset 2

Supplementary Dataset 3

Supplementary Dataset 4

Supplementary Dataset 5

Supplementary Dataset 6

Supplementary Dataset 7

Supplementary Dataset 8

Supplementary Dataset 9

Supplementary Dataset 10

## Data Availability

The data that support the findings of this study are available from the corresponding author upon request.
