# Supplementary material for: Unraveling the Plasmodium vivax sporozoite transcriptional journey from mosquito vector to human host
Source: Sci Rep. 2018 Aug 15;8:12183. doi: 10.1038/s41598-018-30713-1 (PMC6093925; doi:10.1038/s41598-018-30713-1)
Supplement: Supplementary file 1 — Supplementary Information [file 41598_2018_30713_MOESM1_ESM.pdf]

Supplementary Information for:

Unraveling the *Plasmodium vivax* sporozoite transcriptional journey from mosquito vector to human host

Alison Roth[1], Swamy R. Adapa[1], Min Zhang[1], Xiangyun Liao[1], Vishal Saxena[2], Raaven Goffe[1], Suzanne Li[1], Ratawan Ubalee[3], Gagandeep S. Saggu[4], Zarna R. Pala[2], Shilpi Garg[2], Silas Davidson[3], Rays H.Y. Jiang[1*], and John H. Adams[1*]

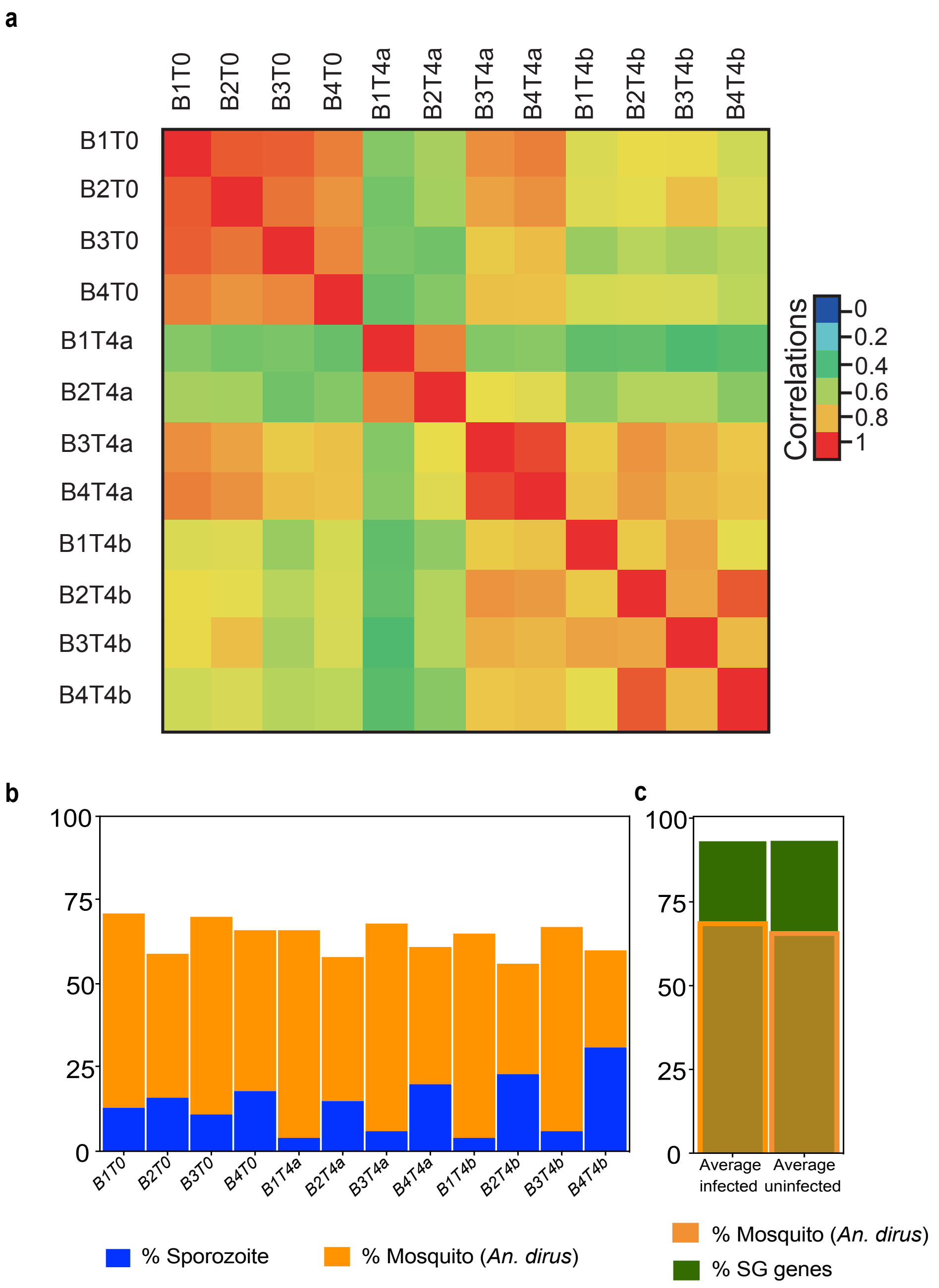

Supplementary Figure S1: Correlation of each RNA-seq sample with the mean of all samples. (a) The correlation coefficients between the gene expression levels in each *P. vivax* sporozoite sample and the mean expression levels of all 48 samples across twelve experimental conditions are reported in the respective graph. The high correlation demonstrated the reliability of detection of RNA-seq. **(b)** Transcript abundance was calculated for all *P. vivax* salivary gland patient isolates in reference for each microenvironment. **(c)** RNA transcripts identified to the *An. dirus* genome were further analyzed with > 93% mapping to salivary gland (SG) associated genes. (B1 = RPMI, B2 = RPMI + 3% BSA, B3 = Schneider's, B4 = Schneider's + 3% BSA and T0 = 0 h post-dissection, T4a = 4 h post-dissection at room temperature, T4b = 4 h post dissection at 37 °C).

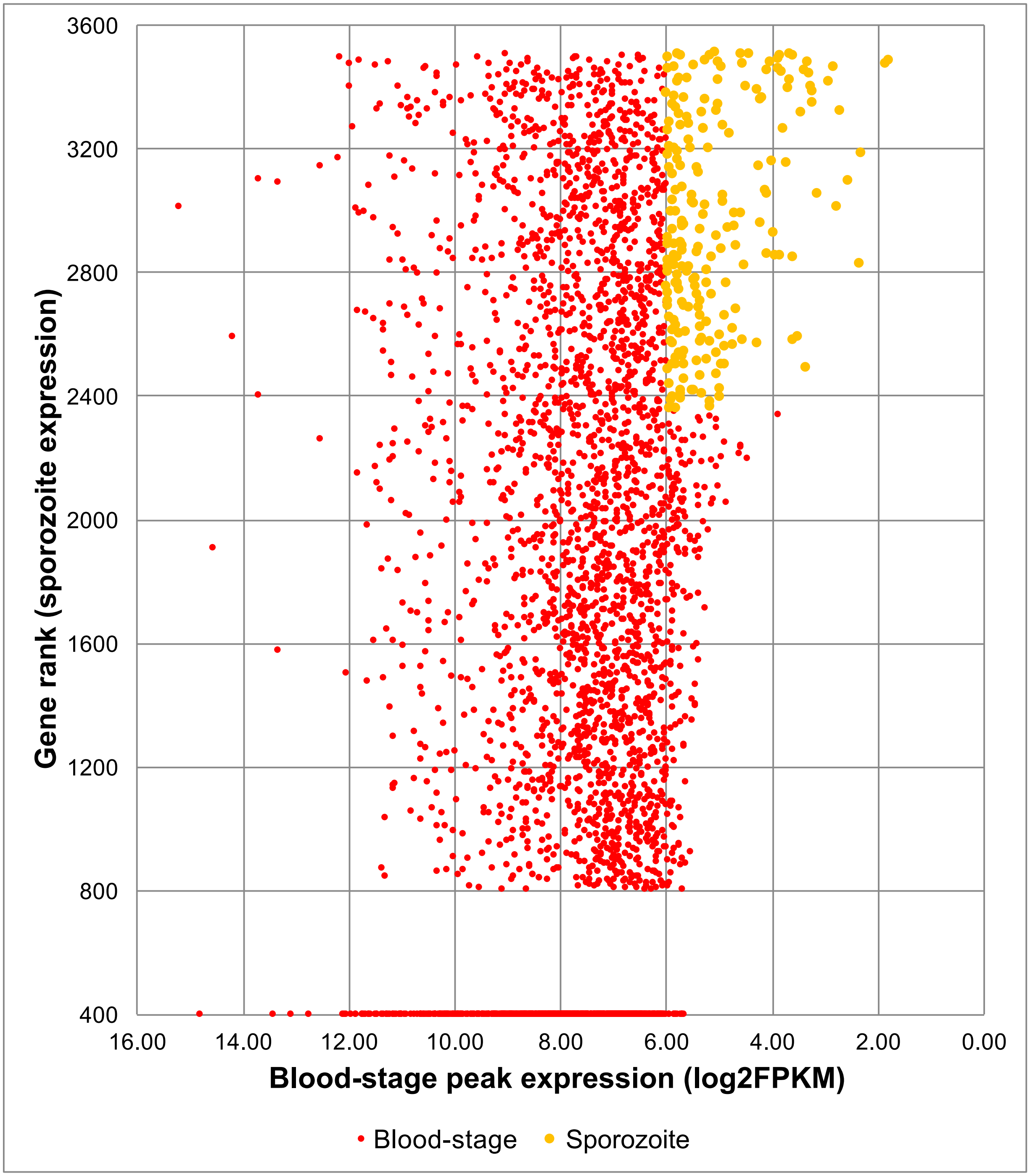

Supplementary Figure S2: Comparison of *Plasmodium vivax* salivary gland sporozoite (PvSGS) transcriptome to *Plasmodium vivax* blood-stage (PvBS). PvSGS RNA-seq samples were compared to published PvBS transcriptome where yellow indicates PvSGS related genes (n = 903) and red indicates PvBS specific genes (n = 2,607).

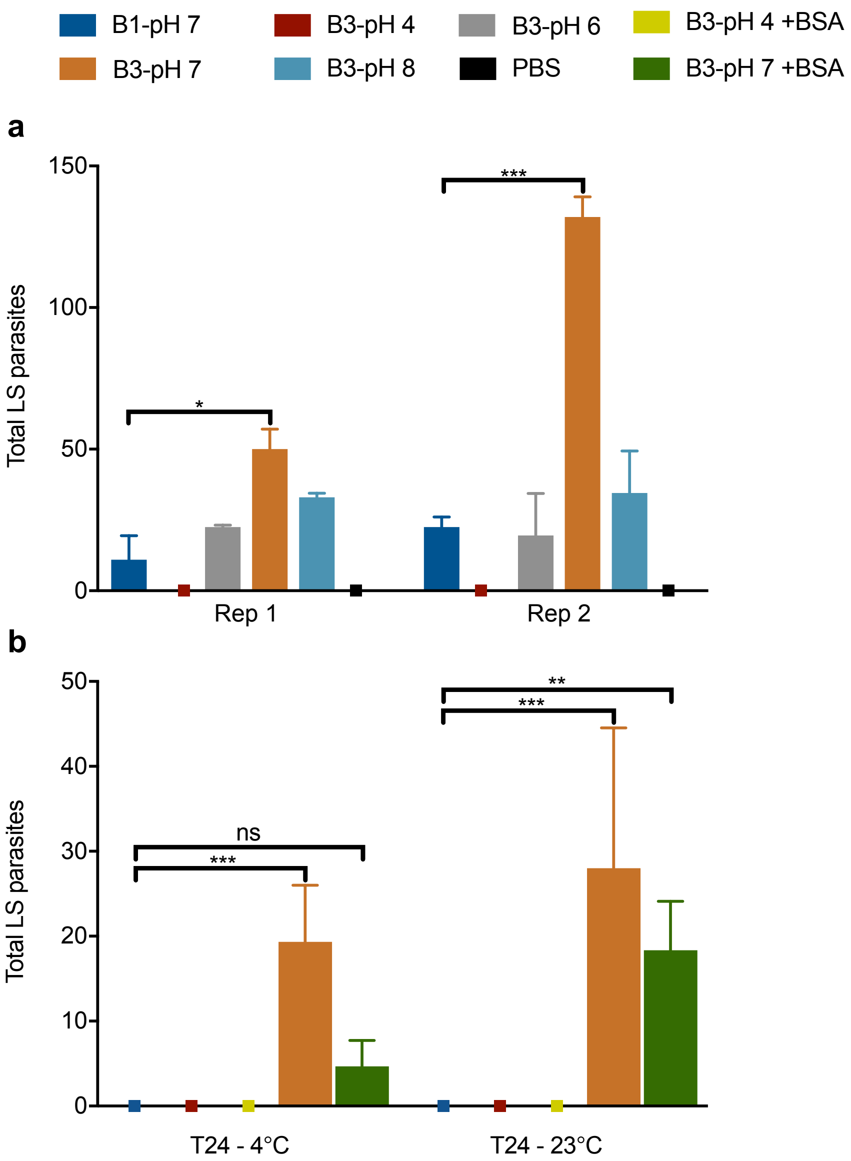

Figure S3: Evaluation of pH effect and time on *P. vivax* salivary gland sporozoites (PvSGSs) measured by liver-stage parasite development in primary human hepatocytes (PHHs). (a) *P. vivax* infected *An. dirus* mosquitoes were shipped to USF and dissected where PvSGSs were isolated into RPMI at pH 7 (B1) or Schneider's (B3) at pH 4, 7, and 8. PvSGSs were diluted in hepatocyte culture media with immediate inoculation into PHHs at 1.8 x 10^4 sporozoites per well. B3 at a pH of 7 showed the highest PHH infection rates. **(b)** PvSGSs were isolated into 5 different media compositions and exposed for 24-hours (T24) at either 4 ºC or RT (room temperature). PvSGSs were then inoculated into PHHs at 1.8 x 10^4 sporozoites per well followed by quantification of liver-stage (LS) parasites developed. Graph bars represent means with s.d for experimental replicates of (n = 3) and biological replicates of (n = 2 (a) and n=1 (b)). Statistical significance was calculated using a two-way ANOVA with Dunnett's multiple comparisons test to B1-pH 7 where statistical significance values are represented as not significant (ns), $P < 0.05$ (*), $P < 0.01$ (**), and $P < 0.0001$ (***).

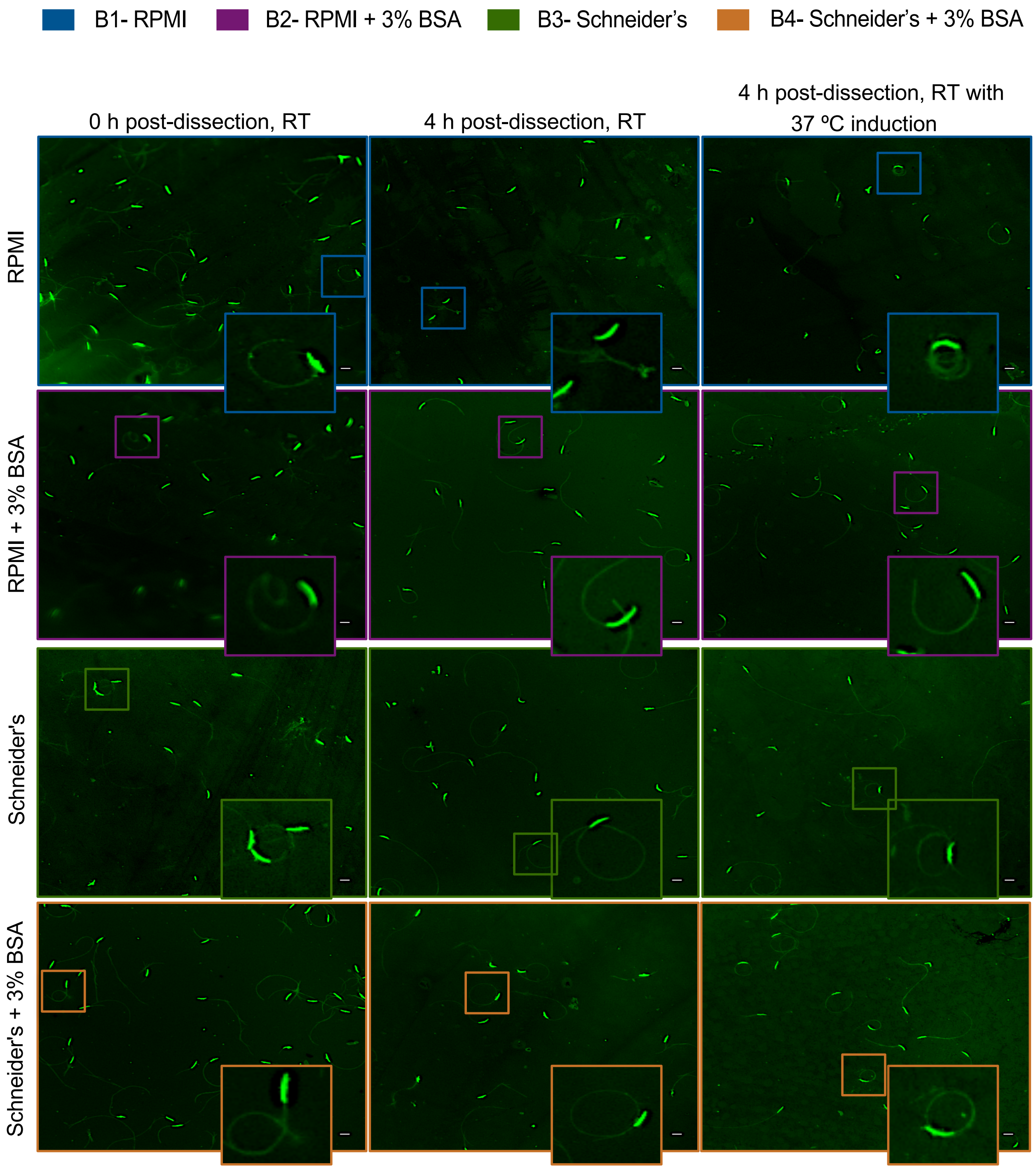

B1- RPMI
B2- RPMI + 3% BSA
B3- Schneider's
B4- Schneider's + 3% BSA
0 h post-dissection, RT
4 h post-dissection, RT
4 h post-dissection, RT with 37 ºC induction
RPMI
RPMI + 3% BSA
Schneider's
Schneider's + 3% BSA

Supplementary Figure S4: Additional time points of *Plasmodium vivax* salivary gland sporozoites (PvSGSs) gliding. Dissected PvSGSs at 0 h post-dissection, RT (room temperature) or at 4 h post-dissection, RT show gliding motility but with reduced circular rotations and a linear trail. When PvSGSs are incubated at 37 ºC for 30min (per requirement of standard motility assay), sporozoites are induced by temperature and exhibit increased motility (B1- RPMI) with more circular rotation. A standard sandwich immunofluorescent assay (IFA) based on the monoclonal anti-circumsporozoite protein (CSP) antibody was used to visualize sporozoite motility where manual quantification (from 10 fields of view or 1,000 total PvSGSs) was used to measure sporozoite metrics (percent gliding, motility path). All images were captured at 20x magnification, 0.74 NA and complimentary to graphical representation in Fig. 3. Scale bars (white) represent 10 µm. (B1 = RPMI, B2 = RPMI + 3% BSA, B3 = Schneider's, B4 = Schneider's + 3% BSA and T0 = 0 h post dissection, T4a = 4 h post incubation at room temperature, T4b = 4 h post incubation at 37 °C).

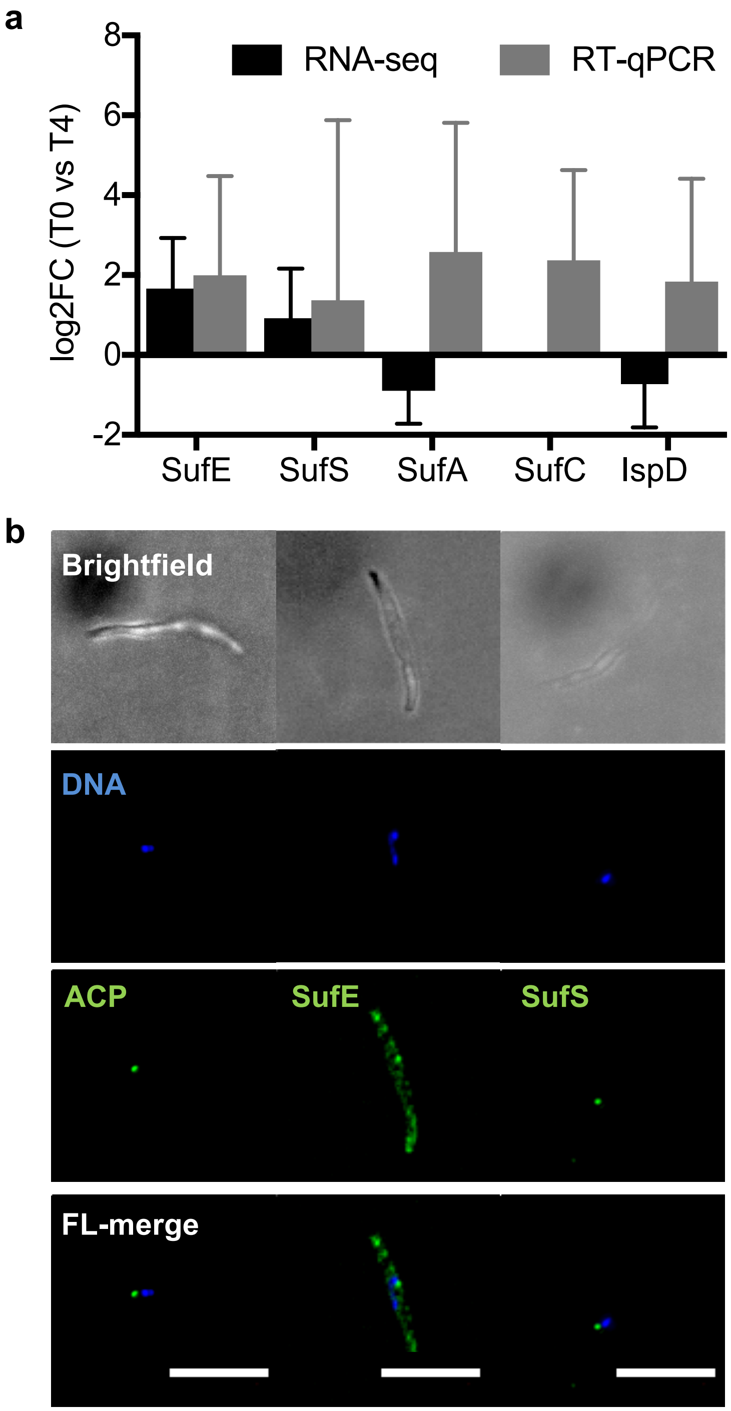

Supplementary Figure S5: Validation of RNA-seq by RT-qPCR and immunofluorescence assay. (a) Comparison of RNA-seq and qPCR log2FC between *P. vivax* salivary gland sporozoites (PvSGSs) 0 h post-dissection (T0) and 4 h post-dissection (T4) samples for select apicoplast genes. **(b).** Immunofluorescent assay (IFA) of PvSGSs stained with apicoplast resident protein acetyl carrier protein (ACP) where Suf E exhibits a diffused pattern in PvSGS and Suf S appears to be localized to the apicoplast.

Supplementary Table S1. Components of experimental mediums.

	**RPMI 1640 (mg/L)**	**Schneider's (mg/L)**
**Inorganic Salts**		
Potassium chloride (KCl)	400	1,600
Sodium chloride (NaCl)	6,000	2,100
Sodium phosphate dibasic (Na_2HPO_4) (anhyd)	800	700
Calcium nitrate ($Ca(NO_3)_2$ $4H_20$)	100	
Magnesium sulfate ($MgSO_4$) (anhyd)	48.84	1,807.221
**Other Components**		
D (+)-Glucose	2,000	2,000
D (+)-Trehalose		2,000
Fumaric acid		60
Succinic acid		60
Yeast extract		2,000
L- (-)-Malic acid		600
α-Ketoglutaric acid		350
Glutathione (reduced)	1	
Phenol red	5.3	
Hypoxanthine	50	
HEPES	10,400	
**Vitamins**		
D-biotin	0.2	
Choline chloride	3	
Folic acid	1	
Niacinamide	1	
p-aminobenzoic acid	1	
D-pantothenic acid ½ Ca	0.25	
Pyridoxine hydrochloride (HCl)	1	
Riboflavin	0.2	
Thiamine hydrochloride	1	
Vitamin B12	0.005	
myo-inositol	35	
**Amino Acids**		
Glycine	10	250
L-Arginine	200	600
L-Asparagine	50	
L-Aspartic acid	20	400
L-Cystine 2HCl	65	26.732
L-Cysteine		60
L-Glutamic acid	20	800
L-Glutamine	300	1,800
L-Histidine	15	400

L-Hydroxyproline	20	
L-Isoleucine	50	150
L-Leucine	50	150
L-Lysine hydrochloride	40	
L-Lysine		1,650
L-Methionine	15	150
L-Phenylalanine	15	
L-Proline	20	1,700
L-Serine	30	250
L-Threonine	20	350
L-Tryptophan	5	100
L-Tyrosine disodium salt dihydrate	28.83	72.02
L-Valine	20	300
β-Alanine		500
Anhydrous referred to as (anhyd)<br>Addition of 3% (w/v) bovine serum albumin (BSA) occurred after media preparation		

This table compares the ingredients between RPMI and Schneider's mediums where the dark grey indicates absent components.

Supplementary Table S2: Primers used for Real-Time expression analysis of sporozoite genes

Gene I.D.	Gene name	Primer name	Primer Sequence
PVP01_1011200	iron-sulfur assembly accessory protein	*Pv*SufA Forward	5'GGA AGG ACG AAA TCG AGG C 3'
		*Pv*SufA Reverse	5'CAG CCG CAC TTT TTA GTG G 3'
PVP01_0312400	cysteine desulfurase, putative	*Pv*SufS Forward	5' GAC TCT ACA TCC ACA ACA G 3'
		*Pv*SufS Reverse	5' CTC CTT CAG CAG CAG AGA 3'
PVP01_0419000	cysteine desulfuration protein SufE, putative	*Pv*SufE Forward	5' GAT CAT TGC GTG GGT AGG 3'
		*Pv*SufE Reverse	5' GTG GAG AAC GCC TTC ATC 3'
PVP01_1335400	FeS assembly ATPase SufC, putative	*Pv*SufC Forward	5' CGG CGT TGA ATA GCC ATC 3'
		*Pv*SufC Reverse	5' CTG AAA CCG TAA TTA ACA GG 3'
PVX_000545	Seryl-tRNA – Synthetase	*PvSTS Forward*	5' CAT CTC CTG AAC GGC ACC AT 3'
		PvSTS Reverse	5' GGT GAA GGG AAT AAA CTC GAC 3'
PVP01_0608000	4-hydroxy-3-methylbut-2-en-1-yl diphosphate synthase, putative	*Pv IspG Forward*	5' GGT TTT CTC CAT GAA GGC TTC CAA C 3'
		Pv IspG Reverse	5' TTG CCC ATC CTT TCA TAT TGC CTC G 3'
PVP01_0207900	2-C-methyl-D-erythritol 4-phosphate cytidylyltransferase, putative	*Pv IspD Forward*	5' GAT TTA TCC CCT TTA TCC GCG 3'
		Pv IspD Reverse	5' GAT AAT GAG AAG GGG AAG TGC 3'

This table shows the primer sequences used for RT-qPCR confirmation for the selected gene I.D.'s.
